# Chromatin-Associated Protein Complexes Link DNA Base J and Transcription Termination in *Leishmania*

**DOI:** 10.1128/mSphere.01204-20

**Published:** 2021-02-24

**Authors:** Bryan C. Jensen, Isabelle Q. Phan, Jacquelyn R. McDonald, Aakash Sur, Mark A. Gillespie, Jeffrey A. Ranish, Marilyn Parsons, Peter J. Myler

**Affiliations:** a Center for Global Infectious Disease Research, Seattle Children’s Research Institute, Seattle, Washington, USA; b Seattle Structural Genomics Center for Infectious Disease, Seattle, Washington, USA; c Department of Biomedical Informatics and Medical Education, University of Washington, Seattle, Washington, USA; d Institute for Systems Biology, Seattle, Washington, USA; e Department of Pediatrics, University of Washington, Seattle, Washington, USA; f Department of Global Health, University of Washington, Seattle, Washington, USA; Indiana University School of Medicine

**Keywords:** *Leishmania*, base J, chromatin remodeling

## Abstract

Unlike most other eukaryotes, *Leishmania* and other trypanosomatid protozoa have largely eschewed transcriptional control of gene expression, relying instead on posttranscriptional regulation of mRNAs derived from polycistronic transcription units (PTUs). In these parasites, a novel modified nucleotide base (β-d-glucopyranosyloxymethyluracil) known as J plays a critical role in ensuring that transcription termination occurs only at the end of each PTU, rather than at the polyadenylation sites of individual genes. To further understand the biology of J-associated processes, we used tandem affinity purification (TAP) tagging and mass spectrometry to reveal proteins that interact with the glucosyltransferase performing the final step in J synthesis. These studies identified four proteins reminiscent of subunits in the PTW/PP1 complex that controls transcription termination in higher eukaryotes. Moreover, bioinformatic analyses identified the DNA-binding subunit of *Leishmania* PTW/PP1 as a novel J-binding protein (JBP3), which is also part of another complex containing proteins with domains suggestive of a role in chromatin modification/remodeling. Additionally, JBP3 associates (albeit transiently and/or indirectly) with the trypanosomatid equivalent of the PAF1 complex involved in the regulation of transcription in other eukaryotes. The downregulation of JBP3 expression levels in *Leishmania* resulted in a substantial increase in transcriptional readthrough at the 3′ end of most PTUs. We propose that JBP3 recruits one or more of these complexes to the J-containing regions at the end of PTUs, where they halt the progression of the RNA polymerase. This decoupling of transcription termination from the splicing of individual genes enables the parasites’ unique reliance on polycistronic transcription and posttranscriptional regulation of gene expression.

**IMPORTANCE** *Leishmania* parasites cause a variety of serious human diseases, with no effective vaccine and emerging resistance to current drug therapy. We have previously shown that a novel DNA base called J is critical for transcription termination at the ends of the polycistronic gene clusters that are a hallmark of *Leishmania* and related trypanosomatids. Here, we describe a new J-binding protein (JBP3) associated with three different protein complexes that are reminiscent of those involved in the control of transcription in other eukaryotes. However, the parasite complexes have been reprogrammed to regulate transcription and gene expression in trypanosomatids differently than in the mammalian hosts, providing new opportunities to develop novel chemotherapeutic treatments against these important pathogens.

## INTRODUCTION

The genus *Leishmania* includes several species of protozoan parasites that cause a spectrum of human diseases, ranging from cutaneous lesions to disfiguring mucocutaneous and lethal visceral leishmaniasis, depending primarily on the species involved. *Leishmania* is transmitted through the bite of the sand fly and belongs to the family Trypanosomatidae, which also includes the vector-borne human pathogens Trypanosoma brucei, the causative agent of human African trypanosomiasis (African sleeping sickness), and Trypanosoma cruzi, the causative agent of Chagas’ disease. Reflecting the ancient divergence of these organisms, the Trypanosomatidae exhibit a myriad of biological differences from “higher” eukaryotes. One major difference is that each chromosome is organized into a small number of polycistronic transcription units (PTUs), which consist of tens to hundreds of protein-coding genes cotranscribed from a single initiation site at the 5′ end of the PTU to a termination site at the 3′ end (1). Interestingly, unlike the operons of prokaryotes, genes within each PTU are not confined to a single pathway or function. Individual genes within the primary transcript are *trans*-spliced by the addition of a 39-nucleotide spliced-leader (SL) miniexon to provide the 5′ cap-4 structure and polyadenylated to form the mature individual mRNAs. As a result of this unique genomic organization, all genes within a PTU are transcribed at the same rate (2, 3). Hence, gene expression must be controlled by posttranscriptional processes such as the splicing/polyadenylation rate, RNA stability, and translational regulation.

A second distinct feature of the Trypanosomatidae (and other Euglenozoa) is that ∼1% of the thymidine bases in the nuclear genome are glucosylated to form the novel nucleotide β-d-glucopyranosyloxymethyluracil, usually referred to as J (4, 5). While the majority of J is localized within telomeric repeat sequences (4, 6–9), chromosome-internal J is found at almost all transcription termination sites (TTSs) (10, 11) and centromeres, which also correspond to the major replication origins on *Leishmania* chromosomes (12, 13). In some trypanosomatids (although not *Leishmania*), J is also found in transcriptionally silent regions containing retrotransposons and/or other repetitive sequences (9). J biosynthesis occurs in two steps, whereby one of two proteins (JBP1 or JBP2) hydroxylates the methyl group of thymidine to form hydroxymethyldeoxyuracil (HmdU), which is subsequently further modified by a glucosyltransferase (HmdUGT) to form J (10, 14). Both JBP1 and JBP2 have an N-terminal oxygenase (Tet_JBP) catalytic domain, but JBP1 contains a central J-binding domain (15), while JBP2 instead contains SNF2_N, ATP-binding, and helicase C-terminal domains that are suggestive of a role in chromatin binding and/or remodeling (16, 17). Null mutants of *JBP1* have not been isolated (despite multiple attempts) in *Leishmania*, suggesting that it is an essential gene (and, hence, that J is required for viability) (18). In contrast, *jbp2*-null mutants have been isolated and shown to have 40% less J than wild-type (WT) parasites (11). Chromosome-internal J was gradually lost during continuous growth of Leishmania tarentolae *jbp2*-null mutants, with a concomitant increase in readthrough transcription at termination sites, suggesting a critical role for J (and JBP2) in transcription termination. Coupled with the identification of a JBP1 recognition motif (19), these data led to the model that JBP1 is responsible for J maintenance after DNA replication by binding to preexisting J on the parental strand and modifying a thymidine 12 nucleotides downstream on the newly synthesized strand. Conversely, JBP2 is proposed to be largely responsible for *de novo* synthesis of J when JBP1 is not able to fully restore J on both strands.

Despite this knowledge of the enzymes involved in J biosynthesis, we currently know little about how J mediates transcription termination (and/or repression of initiation) since the machinery underlying this process has not yet been identified. Here, we have used tandem affinity purification (TAP) tagging and tandem mass spectrometry (MS/MS) to identify a PTW/PP1-like protein complex that interacts with HmdUGT. This complex includes a novel J-binding protein (JBP3) that appears to be essential in *Leishmania.* Transcriptome sequencing (RNA-seq) analysis following conditional downregulation of JBP3 expression shows substantially higher levels of transcriptional readthrough at the 3′ end of most PTUs, suggesting that it plays an important role in transcription termination. While the manuscript was being prepared, Kieft et al. (20) reported similar results in *Leishmania* and T. brucei. Here, we have extended these findings by demonstrating that JBP3 also interacts with another protein complex likely involved in chromatin modification/remodeling as well as, to a lesser degree, with an RNA polymerase II (RNAP II)-associated factor 1 complex (PAF1C)-like complex that likely interacts with RNAP II. Therefore, despite the differences in gene regulation from other eukaryotes, *Leishmania* appears to utilize proteins related to those used for chromatin remodeling and transcriptional regulation in other eukaryotes to provide the molecular machinery that links J to the termination of RNAP II-mediated transcription.

## RESULTS

### Identification of a protein complex containing a novel J-binding protein.

**FIG 1:**
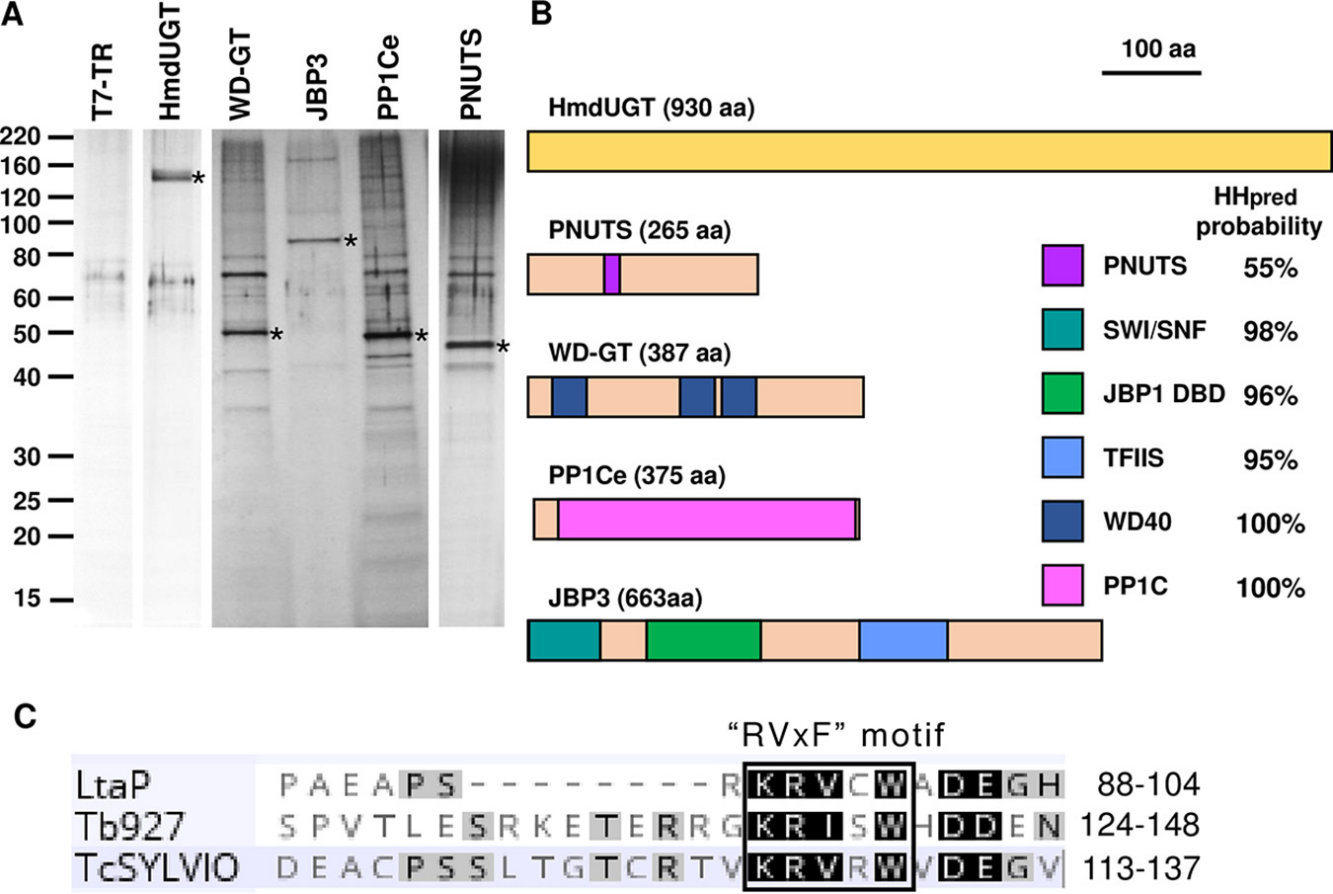
The PJW/PP1 complex. (A) Proteins in the peak fractions from TAPs of HmdUGT and other components of the PJW/PP1 complex (WD-GT, JBP3, PP1Ce, and PNUTS) overexpressed in T7-TR cells were separated by 4 to 20% SDS-PAGE and silver stained. Each lane represents 5% of the total fraction. The TAP-tagged “bait” protein is indicated by an asterisk. The first lane shows the equivalent fraction from a mock purification of control (T7-TR) cells. (B) Schematic representation of key domains in the four proteins (and GT) in the PJW/PP1 complex, as predicted by HHpred analysis. The probability of a match between the *Leishmania* protein and the most similar experimentally determined structure is shown to the right. Potential functions of each domain are discussed in the text. (C) The putative PP1C-interacting domain of L. donovani (LtaP), T. brucei (Tb927), and T. cruzi (TcSYLVIO) PNUTS is shown, and the “RVxF” docking motif (which has a consensus sequence of K/R-K/R-V/I-X-F/W, where X is a residue other than F, I, M, Y, D, or P) (21) is shown at the top. The amino acid (aa) positions for each end of the domain are shown to the right.

To date, only three proteins have been shown to be involved in J biosynthesis: JBP1, JBP2, and HmdUGT (referred to here as GT). To expand the network of proteins important in J biosynthesis and/or function, we used mass spectrometry to identify proteins that copurified with a TAP-tagged GT bait expressed in L. tarentolae. Two separate experiments were performed: the first used extracts from wild-type parasites constitutively expressing the tagged protein, while the second pulldown (and all other TAP-tag experiments) were performed using extracts from a tetracycline (Tet)-induced T7-TR cell line, which overexpresses the tagged protein integrated at the *ODC* locus (see Fig. S1A in the supplemental material). After affinity purification, SDS-PAGE and silver staining confirmed the successful enrichment of the bait protein in the pooled eluates (Fig. 1A). Proteins were identified by liquid chromatography-tandem mass spectrometry (LC-MS/MS), and their (log_2_ fold) enrichment was calculated by comparison to a control (untransfected) parental cell line (see Data Set S1 in the supplemental material for a complete list of all proteins detected in each TAP tag pulldown).

10.1128/mSphere.01204-20.1 FIG S1 Expression of proteins tagged with MHTAP. (A) HmdUGT tagged with MHTAP was transfected into either wild-type (WT) L. tarentolae Parrot-TarII or the L. tarentolae strain T7-TR that expresses both T7 RNA polymerase and a tetracycline (Tet) repressor. Cell lysates were made from transfectants of the WT or the T7-TR strain grown in the presence (+) or absence (−) of Tet. In the absence of Tet, the transgene in T7-TR is repressed, while in its presence, it is induced. Proteins from 5 × 10^6^ cells were separated by SDS-PAGE and transferred to a membrane, and the tagged protein was detected with anti-CBP, which detects the calmodulin-binding domain that is part of MHTAP. (B) Constructs expressing MHTAP-tagged protein were transfected into T7-TR cells, and lysates were generated after treatment with Tet to induce the expression of the transgene. Proteins from 5 × 10^6^ cells were resolved by SDS-PAGE and transferred to a membrane, and MHTAP-tagged protein was detected with anti-CBP. Download FIG S1, PDF file, 0.02 MB. Copyright © 2021 Jensen et al. 2021 Jensen et al. https://creativecommons.org/licenses/by/4.0/ This content is distributed under the terms of the Creative Commons Attribution 4.0 International license.

10.1128/mSphere.01204-20.10 DATA SET S1 Excel spreadsheet showing all proteins identified by mass spectrometry of TAP tag pulldowns. The results from each experiment are shown in different tabs, with color shading used to indicate proteins that show substantial (>32-fold) enrichment over the control. The final tab aggregates the results from all experiments, with color shading used to differentiate proteins in separate complexes. Download Data Set S1, XLSX file, 1.3 MB. Copyright © 2021 Jensen et al. 2021 Jensen et al. https://creativecommons.org/licenses/by/4.0/ This content is distributed under the terms of the Creative Commons Attribution 4.0 International license.

The data revealed substantial (>500-fold) enrichment of five proteins (including the “bait”) in the GT-TAP pulldown: four that were enriched in both replicates and one that was detected only in the first experiment (Table 1). These results suggested that the five proteins form a GT-associated protein complex, which was investigated in more detail, as described below. In addition, six prefoldin subunits were enriched by 50- to 450-fold in one or both replicates (see Table S1 in the supplemental material, which includes all proteins that were substantially enriched in each pulldown). However, since prefoldin likely acts only as a chaperone for one or more proteins of the GT-associated complex, these proteins are not further considered here.

**TABLE 1:** Enrichment^*a*^ of proteins in the PJW/PP1 complex

Gene ID	Gene name	HHpred/InterPro domain(s)	TAP-tagged protein				
			HmdUGT	PP1Ce	PNUTS	JBP3	WD-GT
LtaP36.2450	HmdUGT	None found	14.1	7.2	8.2	10.6	9.2
LtaP15.0230	PP1Ce	Serine/threonine-protein phosphatase	7.4	17.1	12.2	10.9	13.9
LtaP33.1440	PNUTS	PPP1R10/PNUTS	(9.8)	12.5	17.7	10.3	13.2
LtaP36.0380	JBP3	SWI1, J-binding, and N-terminal TFIIS	11.1	12.3	10.3	15.9	12.5
LtaP32.3990	WD-GT	WD40 repeats	9.3	14.8	12.7	12.3	16.3

a Enrichment is expressed as the mean log_2_ fold change compared to control pulldowns. Parentheses indicate that enrichment was observed in only one replicate.

10.1128/mSphere.01204-20.7 TABLE S1 Proteins that copurify with TAP-tagged bait proteins. Only proteins with an average enrichment of >32-fold (>64-fold for PP1Ce and 100-fold for WD-GT) and at least two peptides in both experiments are shown, except for PNUTS and PFDN2 in the HmdUGT pulldowns, CTR9 and CDC73 in the JBP3 pulldowns, and RTF1L in the LEO1 pulldowns. The “bait” in HmdUGT-TAP replicate 1 (Rep 1) was constitutively expressed (in wild-type cells), while the bait in all other experiments was overexpressed by the addition of tetracycline to T7-TR cell lines. The values for HPC-J3C represent the total numbers of peptides detected and the average fold enrichments for 7 different CDSs for this gene, which was misassembled in the LtaP genome. Color shading is used to indicate proteins in the three JBP3-associated complexes described in the legend of Fig. 6. The lighter shading of HmdUGT indicates a more transient PP1 complex/PP1 complex. Download Table S1, PDF file, 0.1 MB. Copyright © 2021 Jensen et al. 2021 Jensen et al. https://creativecommons.org/licenses/by/4.0/ This content is distributed under the terms of the Creative Commons Attribution 4.0 International license.

One of the highly enriched proteins in the GT-TAP pulldown (LtaP15.0230) was annotated in TriTrypDB as a putative protein phosphatase 1 catalytic subunit (PP1C), while the other three (LtaP36.0380, LtaP33.1440, and LtaP32.3990) were all annotated as “hypothetical protein, conserved.” Constructs were made for the expression of TAP-tagged proteins and transfected into the L. tarentolae T7-TR strain. Affinity purification was performed on two (independently generated) cell lines for each version of the TAP-tagged proteins (the results from one replicate of each are shown in Fig. S1B) and analyzed by LC-MS/MS (Data Set 1). The results from these pulldowns (Table 1 and Table S1) show that the four identified proteins described above were all highly enriched (as was GT, albeit to a lesser degree). Thus, we conclude that these proteins form a stable complex, with GT perhaps being more transiently associated than the other four components.

*LtaP15.0230* encodes one of eight isoforms of PP1C found in the L. tarentolae genome. Phylogenetic analysis (Fig. S2) indicates that there are five different clades of PP1C paralogues in trypanosomatids, and PP1Ce (encoded by *LtaP15.0230*) belongs to a clade that lacks any mammalian orthologue. Interestingly, salivarian trypanosomes (including T. brucei) also lack PP1Ce, although it is present in stercorarian trypanosomes (Fig. S2) and the more distantly related kinetoplastids Blechomonas ayalai, Paratrypanosoma confusum, and Bodo saltans (data not shown). TAP tagging of PP1Ce resulted in the copurification of seven proteins with >150-fold enrichment (Table S1). These include GT and the other three components of the complex described above as well as three proteins (encoded by *LtaP05.1290*, *LtaP07.0770*, and *LtaP29.0170*) annotated as protein phosphatase regulatory subunits (PPP1R7/Sds22, PPP1R11/inhibitor 3, and PPP1R2/inhibitor 2, respectively). In higher eukaryotes, PPP1R2 and PPP1R11 use the same “RVxF” docking motif to bind to and inhibit PP1C, while PPPR7 docks at a different site (21). At least some mammalian PP1C proteins form an inactive heterotrimeric complex containing PP1R7 and PPP1R11 (22). Thus, we suggest that PP1Ce forms at least three separate complexes: one with both PPP1R7 and PPP1R11, a second with PPP1R2 (which may or may not contain PPP1R7), and the third being the GT-associated complex, as described below.

10.1128/mSphere.01204-20.2 FIG S2 Phylogenetic tree for PP1C proteins from selected kinetoplastid species in comparison to proteins from human and Saccharomyces cerevisiae. Sequences were aligned using Muscle and viewed in Geneious (Geneious Prime 11.0.5). Genetic distances were calculated using the Jukes-Cantor method, and trees were constructed using neighbor joining. The five clades of PP1C are shown, with genes from *Leishmania* (green dots), T. brucei (blue dots), T. cruzi (red dots), and human (black dots) indicated. Genus and species for proteins used in the alignments are Crithidia fasciculata (Gene IDs CFAC1_060020300, CFAC1_300013300, CFAC1_270060100, CFAC1_290037400, CFAC1_290037500, CFAC1_290037600, CFAC1_290037700, CFAC1_290037800, and CFAC1_290038200), human (UniProt gene identifiers PP1A_HUMAN, PP1B_HUMAN, and PP1G_HUMAN), Leishmania major (Gene IDs LmjF.15.0220, LmjF.28.0690, LmjF.31.2630, LmjF.34.0780, LmjF.34.0790, LmjF.34.0800, LmjF.34.0810, and LmjF.34.0850), Leishmania tarentolae (Gene IDs LtaP15.0230, LtaP28.0710, LtaP31.3050, LtaP34.0890, LtaP34.0920, LtaP34.0930, LtaP34.0940, and LtaP34.0980), Leptomonas seymouri (Gene IDs Lsey_0031_0170, Lsey_0328_0090, Lsey_0221_0060, and Lsey_0565_0010), Trypanosoma brucei (Gene IDs Tb927.4.3560, Tb927.4.3610, Tb927.4.3620, Tb927.4.3630, Tb927.4.3640, Tb927.4.5030, Tb927.8.7390, and Tb927.11.8090), Trypanosoma cruzi (Gene IDs TcCLB.506739.130, TcCLB.508815.110, TcCLB.506201.30, TcCLB.506201.70, TcCLB.506201.80, TcCLB.507757.50, and TcCLB.509633.50), Trypanosoma vivax (Gene IDs TvY486_0019980, TvY486_0403330, TvY486_0403390, TvY486_0806880, and TvY486_1108810), and Trypanosoma grayi (Gene IDs DQ04_02191050, DQ04_11101010, DQ04_01081070, DQ04_02221000, DQ04_13331010, and DQ04_16591000). The four L. tarentolae clade C genes are incomplete in the genome deposited at TriTrypDB, with each gene harboring internal ambiguous bases. Full-length copies of these genes derived from our in-house genome for L. tarentolae were used in this figure. Gene IDs for these genes were assigned based on alignment with the incomplete copies available at TriTrypDB. Download FIG S2, PDF file, 0.1 MB. Copyright © 2021 Jensen et al. 2021 Jensen et al. https://creativecommons.org/licenses/by/4.0/ This content is distributed under the terms of the Creative Commons Attribution 4.0 International license.

We performed a series of bioinformatic analyses to identify domains and/or motifs that might provide hints as to the function of the three proteins of the GT-associated complex that lack a functional description. BLASTP and InterProScan searches showed high-confidence matches only to orthologues in other trypanosomatids with no informative domains identified. However, HHpred analysis, which detects remote protein homology by using hidden Markov models and structure prediction (23), revealed a number of structural matches, which are summarized in Fig. 1B and shown in detail in Fig. S3. HHpred predicts that the central portion (residues 88 to 105) of LtaP33.1440 contains structural similarity to a portion of human serine/threonine-protein phosphatase 1 regulatory subunit 10 (PPP1R10), also known as PNUTS (for PP1 nuclear targeting subunit) (24–27). The trypanosomatid protein is much smaller (264 versus 940 amino acids) than mammalian PNUTS, with the sequence and structural similarities restricted to the central region described above (Fig. S3A). However, this region contains the RVxF sequence motif (Fig. 1C) noted above, which is present in most inhibitors and responsible for interaction with PP1C (28). Therefore, in deference to precedent in the field (20), we refer to this protein as PNUTS despite the lack of an obvious nuclear localization signal. TAP-tagging of PNUTS showed significant enrichment of the four other components of the GT-associated protein complex and no other proteins (Table S1).

10.1128/mSphere.01204-20.3 FIG S3 Sequence analysis of identified proteins using HHpred. The schematics for each predicted protein show the length of the complete protein plus coordinates where the top hit of the protein database (PDB) aligned. Hits (predicted to be structurally similar to the query) are color-coded, with high-probability hits in red and low-probability hits in blue and black. Below the schematic showing the best hits are the HHpred descriptions of the corresponding proteins and similarities. This includes the PDB accession number and chain designation (Hit); a description of the PDB entry, including related PDB entries (Name); the probability of the hit based on the hidden Markov model (Probability); the probability of the match in an unrelated database (E value); the score for the secondary-structure prediction (SS); the number of amino acids aligned (Col); and the total length of the target in PDB entry (Target Length). The documentation for HHpred considers “probability” the most important criterion, with hits of >50% being considered significant. Download FIG S3, PDF file, 0.4 MB. Copyright © 2021 Jensen et al. 2021 Jensen et al. https://creativecommons.org/licenses/by/4.0/ This content is distributed under the terms of the Creative Commons Attribution 4.0 International license.

BLASTP searches of LtaP32.3990 returned matches to orthologues in other trypanosomatids as well as WD40 repeats in proteins from several other organisms, while HHpred analysis (Fig. S3B) identified at least three WD40 repeats. Therefore, we refer to this protein as WD-GT, to distinguish it from the numerous other WD40 repeat-containing proteins in *Leishmania*. TAP-tagged WD-GT pulled down the four other components of the GT-associated protein complex as well as a number of chaperone-associated proteins, including prefoldin, T-complex, and heat shock proteins (Table S1).

**FIG 2:**
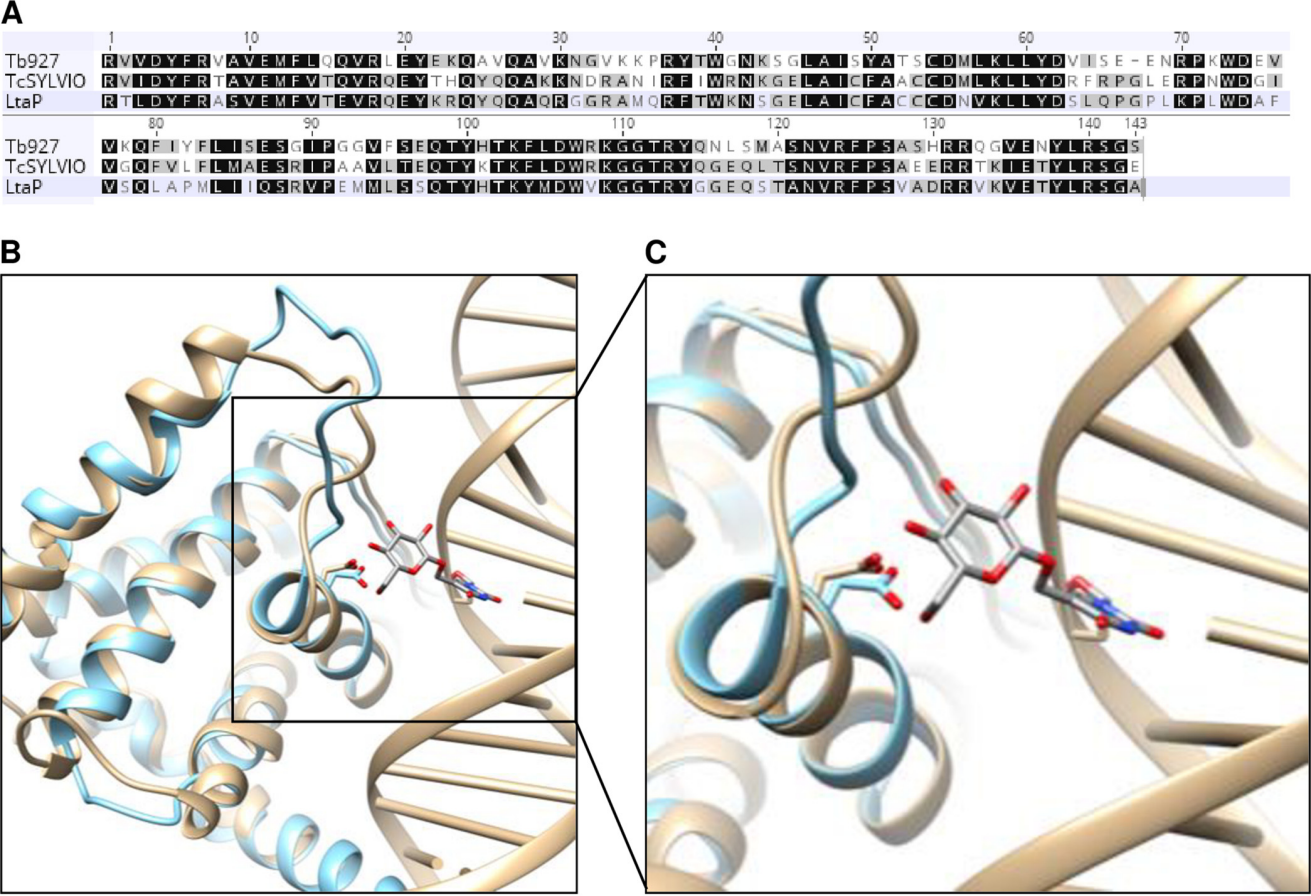
Modeling of the JBP3 DNA-binding domain. (A) Sequence alignment of the putative JBP3 J-binding domains from the T. brucei EATRO927 strain, the T. cruzi Silvio strain, and the L. tarentolae Parrot strain. Residues that are identical or conservatively replaced in all three species are shaded black, while those that are identical or conserved in two species are shaded gray. (B) The structure of the DNA-binding domain from JBP3 (light blue) was modeled using RosettaCM against the J-binding domain of JBP1 (tan) from PDB accession number 2XSE. The interaction between the conserved aspartic acid residue (Asp_525_ in JBP1 and Asp_241_ in JBP3) and the glucose of base J is shown. (C) Higher-resolution view of the interaction between the conserved aspartate of both proteins and base J.

HHpred analysis of LtaP36.0380 revealed three separate domains with structural similarity to different proteins (Fig. S3C). The N-terminal domain (residues 2 to 86) is similar to the central portion of the SWI1 subunit of the Saccharomyces cerevisiae SWI/SNF chromatin remodeling complex, while the C-terminal domain (residues 384 to 485) is related to the N-terminal TFIIS domain of mammalian PNUTS. Most importantly, the central portion (residues 137 to 269) is predicted to have substantial structural similarity to the DNA-binding domain (DBD) of JBP1, and *in silico* folding of this region revealed conservation of the functional signature D-(W/F/Y)-x-x-GGTRY motif present in all trypanosomatid JBP1 proteins (Fig. 2A). In addition, a structural model of the DBD from LtaP36.0380 contains a binding pocket large enough to accommodate the glucose ring of J (Fig. 2B and C), and preliminary experiments indicate that it binds preferentially to J (Anastasis Perrakis, NKI, Amsterdam, Netherlands, personal communication). While the manuscript was in preparation, this J-binding function of LtaP36.0380 was experimentally confirmed by others (20), so the protein was renamed J-binding protein 3 (JBP3).

The molecular characteristics of the four proteins identified in the GT pulldowns, a PP1 catalytic subunit (PP1Ce), a predicted PP1 regulatory protein (PNUTS), a WD40 repeat protein (WD-GT), and a DNA-binding protein (JBP3), are highly reminiscent of the components of the mammalian PTW/PP1 complex. This complex, which contains PP1C, PNUTS, WDR82, and the DNA-binding protein TOX4, has a role in controlling chromatin structure (27, 29). Importantly, the mammalian PTW/PP1 complex was recently found to negatively regulate the RNAP II elongation rate by dephosphorylating the transcription elongation factor Spt5, leading to transcription termination at polyadenylation sites (30). Thus, our results indicate that GT associates with a PTW/PP1-like complex in *Leishmania* (which we refer to as PJW/PP1), wherein JBP3 replaces the DNA-binding function of TOX4.

### JBP3 is part of another chromatin remodeling complex.

While tandem affinity purification of TAP-tagged JBP3 showed >256-fold enrichment of the PJW/PP1 complex proteins (PP1Ce, PNUTS, WD-GT, and GT) (Table S1), another four proteins (encoded by *LtaP35.2400*, *LtaP28.2640*, *LtaP12.0900*, and *LtaP14.0150*) were >3,000-fold enriched (Table 2). BLASTP analyses of these proteins failed to reveal convincing matches to anything other than orthologues in other trypanosomatids, and InterProScan showed no matches using the default parameters. However, LtaP35.2400 is annotated as a “SET domain-containing protein, putative,” in TriTrypDB, and HHpred analysis revealed that the N-terminal region (amino acids 70 to 227) contains structural similarity to SET domain-containing proteins, while the central portion (residues 355 to 385) shows weaker similarity to C4-type zinc finger domains from several unrelated proteins (Fig. S3D). SET domains, which are usually involved in binding to and/or methylation of histones (31), are also present in several other *Leishmania* proteins, so we have named this protein SET-J3C to distinguish it from the others. HHpred analysis of LtaP14.0150 showed structural similarity to chromatin organization modifier (Chromo) domains (32, 33) from numerous eukaryotic proteins at its N terminus (amino acids 1 to 55) and weak similarity to Chromo shadow domains at the C terminus (34) (Fig. S3E). Therefore, we dubbed this protein Chromo-J3C and predict that it may be involved in the recognition of methylated lysine residues on histone tails. LtaP28.2640 is annotated as a “hypothetical protein, conserved,” but residues 187 to 227 also show structural similarity to the Chromo shadow domain (Fig. S3F), and so we called it CS-J3C. The *LtaP12.0900* gene is misassembled in the L. tarentolae reference genome, so we used full-length orthologues from other *Leishmania* genomes for subsequent analyses. However, BLASTP, InterProScan, and HHpred analyses were uninformative, so we called this protein HPC-J3C (for hypothetical protein, conserved in J3C). Phylogenetic analysis showed that HPC-J3C has poor sequence conservation, even in other trypanosomatids, with orthologues in other genera being shorter than in *Leishmania*. HHpred analysis of the T. brucei orthologue (Tb927.1.4250) showed structural similarity at the C terminus to subunits from several large protein complexes involved in a variety of processes, including histone remodeling (Fig. S3G).

**TABLE 2:** Enrichment^*a*^ of proteins in the JBP3-associated chromatin remodeling complex

Gene ID	Gene name	HHpred/InterPro domain(s)	TAP-tagged protein		
			JBP3	J3C-Chromo	J3C-CS
LtaP36.0380	JBP3	SWI1, J-binding, and N-terminal TFIIS	15.9	7.9	7.8
LtaP35.2400	SET-J3C	SET and C4-type Zn finger	12.8	8.2	9.4
LtaP14.0150	Chromo-J3C	Chromo and Chromo shadow	11.7	11.6	8.8
LtaP28.2640	CS-J3C	Chromo shadow	12.5	9.8	11.8
LtaP12.0900	HPC-J3C	None found	12.1	9.8	6.3

a Enrichment is expressed as the mean log_2_ fold change compared to control pulldowns.

**FIG 3:**
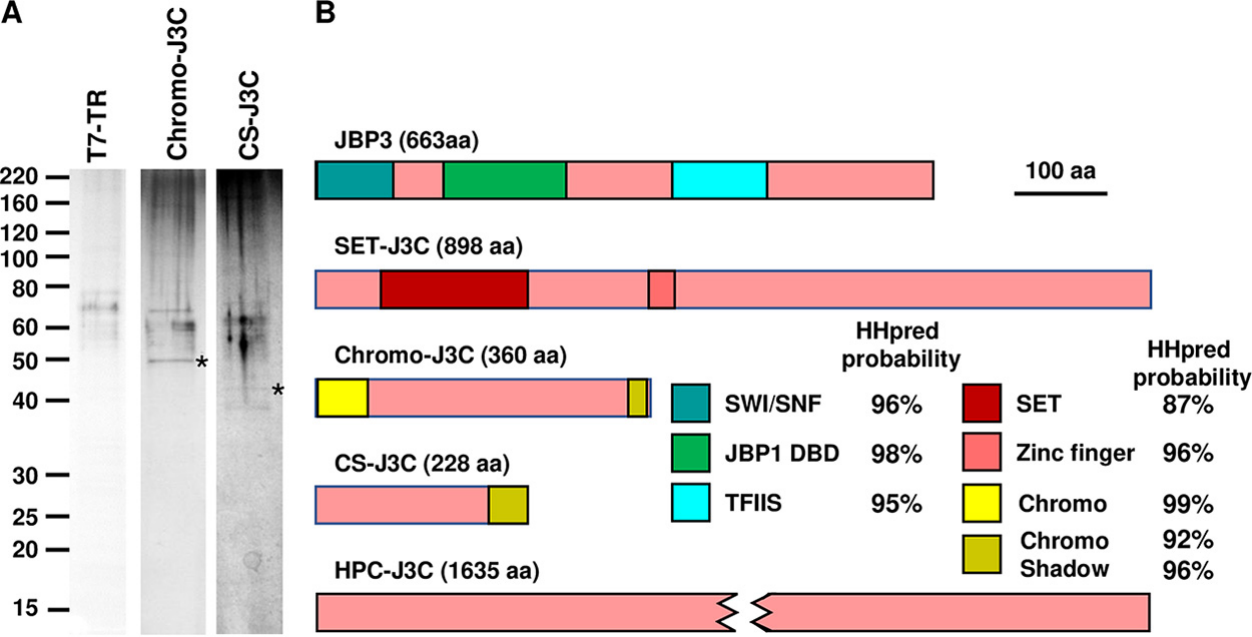
The JBP3-associated chromatin complex. (A) Proteins that copurify with TAP-tagged Chromo-J3C and CS-J3C overexpressed in T7-TR cells were analyzed by SDS-PAGE and silver staining as described in the legend of Fig. 1. (B) Schematic representation showing the key domains of the five proteins that copurified in the JBP3-associated chromatin (J3C) complex.

To confirm the association of these proteins with JBP3 (and each other), we transfected TAP-tagged versions of them into L. tarentolae T7-TR. Unfortunately, cloning of *HPC-J3C* failed because of errors in the genome sequence (see above), and transfectants containing the *SET-J3C* construct did not express the tagged protein (perhaps because overexpression was deleterious for cell growth), but the Chromo-J3C and CS-J3C transfectants expressed tagged proteins of the expected size (Fig. S1B), enabling affinity purification (Fig. 3A). Subsequent mass spectrometric analysis of copurifying proteins showed that Chromo-J3C and CS-J3C pulldowns enriched JBP3 and the same JBP3-associated proteins (Table 2 and Table S1), indicating that they form a separate JBP3-containing complex. Bioinformatic analyses (described above) suggest that the proteins in this complex are likely associated with chromatin modification and/or remodeling, prompting us to call it the JBP3-associated chromatin complex (abbreviated J3C).

### JBP3 interacts with PAF1C.

**FIG 4:**
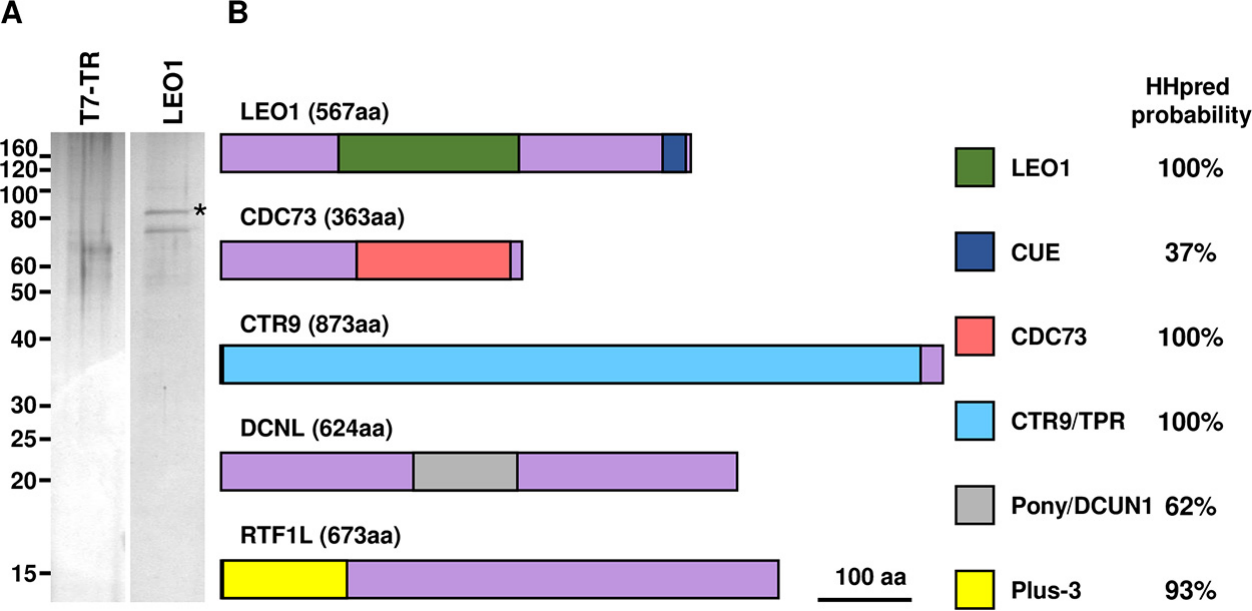
The PAF1C-like complex. (A) Proteins that copurified with TAP-tagged LEO1 overexpressed in T7-TR cells were analyzed by SDS-PAGE and silver staining as described in the legend of Fig. 1. (B) Schematic representation showing the key domains of five components of the PAF1C-L complex and their probability scores from HHpred analysis.

In addition to the components of the PJW/PP1 and J3C complexes, two other proteins (encoded by *LtaP35.2870* and *LtaP29.1270*) were substantially enriched (>110-fold and >23-fold, respectively) in both JBP3 TAP tag experiments (Table 3 and Table S1). LtaP35.2870 is annotated in TriTrypDB as “RNA polymerase-associated protein LEO1, putative,” and this homology was confirmed by HHpred analyses (Fig. S3H). LEO1 is a subunit of the RNAP II-associated factor 1 complex (PAF1C), which facilitates transcription elongation by regulating chromatin modification (35–37). Interestingly, mass spectrometric analysis of proteins that copurified with TAP-tagged LEO1 (Fig. 4A and Table 3) did not detect JBP3 but identified three proteins (encoded by *LtaP36.4090*, *LtaP29.2750*, and *LtaP29.1270*) that were enriched >630-fold in both experiments (Table S1). The first two were also enriched >50-fold in one of the two JBP3 TAP tag experiments (Table S1) and are obvious homologues of PAF1C subunits. HHpred analyses showed that LtaP36.4090 contains the Ras-like fold characteristic of the C-terminal domain of the CDC73 subunit of PAF1C (Fig. S3I) and is annotated as such in TriTrypDB. LtaP29.2750 contains several tetratricopeptide repeat (TPR) domains implicated in protein-protein interactions and shows considerable overall similarity to the CTR9 subunit of PAF1C (Fig. S3J). Functional studies of the T. brucei CTR9 orthologue (Tb927.3.3220) indicated that it is essential for parasite survival, and the depletion of its mRNA reduced the expression of many genes involved in the regulation of mRNA levels (38). A fourth protein (LtaP29.1270) that was also substantially enriched in the TAP-tagged JBP3 experiments is not an obvious orthologue of any known PAF1C subunit. This protein is annotated as a “hypothetical protein, conserved,” in TriTrypDB, but HHpred analysis (Fig. S3K) revealed a central domain (amino acids 232 to 358) with structural similarity to the PONY/DCUN1 domain found in DCN (defective in cullin neddylation) proteins that are involved in the regulation of ubiquitin ligation cascades (39). Our results (Table 3) suggest that LtaP29.1270, which we refer to as DCNL (DCN-like) here, forms an integral part (along with LEO1, CDC73, and CTRL) of a PAF1C-like (PAF1C-L) complex in *Leishmania*. The lack of significant reciprocal enrichment of JBP3 in the LEO1-TAP pulldowns (this study) and its absence from the CTR9-TAP pulldowns in T. brucei (38) suggest a transient and/or indirect association of JBP3 with PAF1C-L.

**TABLE 3:** Enrichment^*a*^ of proteins in the PAF1C-L complex

Gene ID	Gene name	HHpred/InterPro domain	TAP-tagged protein	
			JBP3	LEO1
LtaP35.2870	LEO1	Leo1-like protein	7.4	16.8
LtaP29.1270	DCNL	PONY/DCUN1 domain	5.4	13.9
LtaP36.4090	CDC73	Cell division control protein 73 (C terminal)	(5.7)	10.4
LtaP29.2750	CTR9	RNA polymerase-associated protein Ctr9	(6.2)	9.5
LtaP14.0860	RTF1L	Plus-3 domain	0.6	3.8

a Enrichment is expressed as the mean log_2_ fold change compared to control pulldowns. Parentheses indicate that enrichment was observed in only one replicate.

TAP tagging of T. brucei CTR9 by others (38) revealed the same constellation of PAF1C-L subunits (LEO1, CDC73, and DCNL) as well as an additional protein (Tb927.7.4030). Close examination of our results revealed that the *Leishmania* orthologue (LtaP14.0860) of Tb927.7.4030 is also enriched >10-fold in both LEO1 TAP tag experiments (Table 3 and Table S1). While this protein is annotated as a “hypothetical protein, conserved,” in TriTrypDB, HHpred analysis revealed N-terminal (amino acids 2 to 152) structural similarity to the Plus-3 domain of human RTF1 (Fig. S3L), a component of human and yeast PAF1C. Thus, LtaP14.0860 (which we have dubbed RTF1L) is likely the functional equivalent of RTF1, although in *Leishmania*, it may be less tightly associated with PAF1C-L.

### Depletion of JBP3 causes defects in transcription termination.

**FIG 5:**
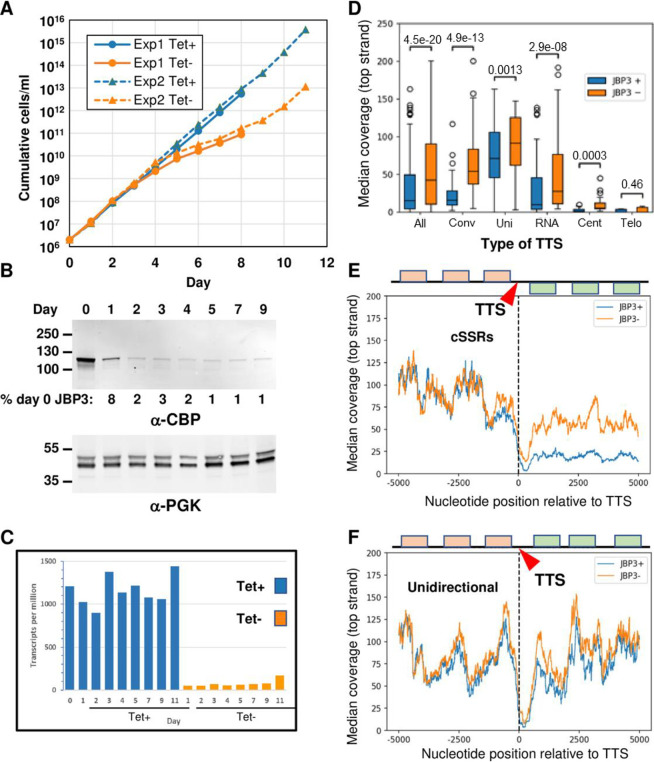
Depletion of JPB3 results in readthrough at transcription termination sites. (A) Growth analysis. Two independently generated L. tarentolae clones lacking endogenous *JBP3* but containing a Tet-regulated TAP-tagged *JBP3* gene were grown in the presence or absence of Tet. The numbers on the *y* axis are corrected for dilution during subculturing. The solid lines show a clone where the *JBP3* genes were replaced by *pac* (and grown in the presence of puromycin), while the dotted lines show a clone where the *JBP3* genes were replaced by *neo* (and grown in G418). Exp1 and Exp2 refer to experiments 1 and 2, respectively. (B) The level of TAP-tagged JBP3 expressed by the T7-TR/JBP3-MHTAP/Δ*jbp3*::*neo* clone grown in the absence of Tet was monitored by Western blotting using antibodies against the calmodulin-binding peptide (CBP) of the TAP tag. Antibodies against phosphoglycerate kinase (PGK) served as a loading control. The percent JBP3 levels in comparison to day 0 are shown below the anti-CBD blot. (C) *JBP3-TAP* mRNA levels for the T7-TR/JB3-MHTAP/Δ*jbp3*::*neo* clone grown in the presence or absence of Tet for the number of days indicated. mRNA levels are expressed as transcripts per million as determined by RNA-seq analysis using Geneious. (D) Box-and-whisker plots showing the median top strand coverage in the 5-kb region downstream of all 192 TTSs (All). Separate plots are shown for the 46 TTSs at cSSRs (Conv), 30 TTSs between head-to-tail PTUs (Uni), 39 TTSs immediately upstream of one or more RNA genes (RNA), 21 TTSs adjacent to a centromere (Cent), and 56 TTSs at telomeres (Telo). (E) Median top strand coverage at each nucleotide position in the 10 kb surrounding the 46 TTSs at cSSRs. The schematic represents the protein-coding genes associated with each strand at an “average” convergent TTS (cTTS). The second PTU at each cSSR is reoriented so that the genes are represented on the top strand. (F) Median top strand coverage at each nucleotide position in the 10 kb surrounding the 30 TTSs between unidirectional (head-to-tail) PTUs. The schematic represents the protein-coding genes associated with each strand at an “average” unidirectional TTS (uTTS).

The results of the TAP-tag experiments presented above suggest that JBP3 is an integral part of two protein complexes (PJW/PP1 and J3C) and interacts, possibly indirectly, with another (PAF1C-L). Since similar complexes have been associated with chromatin modification/remodeling and regulation of transcription in other organisms, we postulated that JBP3 may mediate transcription termination in *Leishmania*. To test this hypothesis, we used CRISPR/Cas9 (40) to delete *JBP3* in the L. tarentolae cell line bearing a tetracycline (Tet)-regulated copy of *JBP3-TAP* (see above). We were able to delete both endogenous copies of *JBP3* only when *JBP3-TAP* expression was induced with Tet, suggesting that it is essential in *Leishmania*. To interrogate the effect of JBP3 depletion, we grew two stable transfectants lacking both endogenous copies of *JBP3* (but containing a Tet-regulated copy of *JBP3-TAP*) for 8 to 11 days in the presence or absence of tetracycline (Fig. 5A). Cells grown in the presence of drug maintained a constant growth rate (with a generation time of ∼9 h) over the length of the experiment, while the growth rate in the absence of drug decreased after day 3, with the generation time increasing to >20 h on day 6, before returning to almost the parental wild-type rate after day 10 (Fig. 5A). JBP3-TAP protein levels decreased markedly during the first day after the removal of tetracycline, dropping to ∼2% of the initial level by day 2 (Fig. 5B).

To assess the role of JBP3 in mRNA expression and transcription termination, RNA was isolated daily from the cells after Tet withdrawal and used to generate strand-specific RNA-seq libraries. Illumina sequencing reads were mapped to the L. tarentolae reference genome, and normalized read counts were calculated for every gene. Differential expression analysis revealed that *JBP3-TAP* mRNA levels were ∼20-fold lower in the absence of Tet (Fig. 5C). Interestingly, there was a marked increase in the *JBP3-TAP* mRNA levels in the day 11 sample lacking Tet (Tet^−^ sample), coincident with the resumption of normal growth. Consequently, this sample was excluded from subsequent analyses, along with the day 1 Tet^−^ samples (since JBP3 protein was still present at ∼8% of the initial level). Further analysis (using the DESeq2 module of Geneious) revealed that 17 genes had significantly higher mRNA abundances (>2-fold; *P* < 0.001) in the remaining Tet^−^ samples, which all had low JBP3 protein levels (Table S2). Interestingly, 14 of these genes are located adjacent (or close) to transcription termination sites (TTSs). Indeed, 34 of the 50 most upregulated genes are located near TTSs, with 24 of these located at convergent strand-switch regions (cSSRs) where the 3′ termini of two PTUs converge.

10.1128/mSphere.01204-20.8 TABLE S2 Genes with increased mRNA levels after depletion of JBP3. The 50 genes showing the largest average (between replicates 1 and 2) significant (*P* < 0.001) increases in RNA abundance, as determined by RNA-seq analysis, are shown in decreasing order of log_2_ fold changes (FC). Different shading intensities indicate those genes increasing by more than 4-fold (dark), 2-fold (medium), or 1.5-fold (light). The “locus type” indicates whether the genes were immediately adjacent to (or within 20 kb of) convergent, unidirectional, or telomeric transcription termination sites (cTTS, uTTS, and tTTS, respectively) or located within the central region of a polycistronic transcription unit (PTU-internal). TTSs located at centromeres are indicated by “-Cen.” Download Table S2, PDF file, 0.1 MB. Copyright © 2021 Jensen et al. 2021 Jensen et al. https://creativecommons.org/licenses/by/4.0/ This content is distributed under the terms of the Creative Commons Attribution 4.0 International license.

To further characterize these increases in RNA abundance, we analyzed the read coverage for 5 kb on either side of all 192 TTSs in the L. tarentolae genome. As expected, when JBP3 is expressed (samples in the presence of Tet [Tet^+^ samples]) the median-normalized coverage on the top (coding) strand decreased sharply downstream of the TTS (Fig. S4A). However, in samples with very low JBP3 protein levels (day 2 to 9 Tet^−^), the read coverage downstream of the TTS was significantly higher, suggesting that reduction of JBP3 resulted in substantial transcriptional readthrough. Importantly, this increase in readthrough transcription did not occur to the same extent at different types of TTSs (Fig. 5D). It was most pronounced at the 23 noncentromeric cSSRs without RNA genes (Fig. 5E and Fig. S4B), where the transcript abundance was almost as high downstream of the TTS as it was upstream. There was also a significant increase in readthrough transcription downstream of the TTS between unidirectionally (head-to-tail) oriented PTUs (Fig. 5F and Fig. S4C), although it was more subtle since the gap between each PTU is small. Conversely, there was only a small increase in readthrough at TTSs upstream of RNA genes transcribed by RNAP III (Fig. S4D) and essentially no readthrough at centromeres (Fig. S4E) or telomeres (Fig. S4F). Analysis of bottom (noncoding) strand transcripts revealed no significant differences between Tet^+^ and Tet^−^ samples (Fig. S4, left), except at cSSRs, where readthrough from the second PTU results in a substantial increase in antisense transcripts upstream of the TTS (Fig. S4B) due to readthrough from the convergent downstream PTU. A similar analysis of transcript abundance surrounding transcription start sites (TSSs) revealed no significant changes due to JBP3 depletion, except for a small increase in top (coding) strand coverage when PTUs were oriented unidirectionally (Fig. S5C and G), presumably due to readthrough from the preceding PTU. Importantly, there was little or no increase in bottom (noncoding) strand coverage upstream of most TSSs.

10.1128/mSphere.01204-20.4 FIG S4 Effect of depletion of JBP3 on transcription termination. The normalized read counts are shown for the 10 kb surrounding TTSs for T7-TR/JBP3-MHTAP/Δ*jbp3*::*neo* grown in the presence (JBP3+) (blue line) or absence (JBP3−) (orange line) of Tet. Plots are oriented such that transcription proceeds from the left and terminates at “0,” with the top strand being the coding strand on the left side of the TTS. The normalized read counts are shown. Panels on the left depict reads mapping to the top strand, and panels on the right depict reads mapping to the bottom strand. (A) All TTSs. (B) TTSs in cSSRs lacking either an RNA gene or a centromere. (C) Unidirectional (“head-to-tail”) TTSs. (D) TTSs immediately upstream of an RNA gene. (E) TTSs adjacent to a centromere. (F) TTSs adjacent to a telomere. (G) Box-and-whisker plots showing the median coverage in the 5-kb region downstream of all TTSs (All), TTSs at cSSRs (Conv), TTSs between head-to-tail PTUs (Uni), TTSs immediately upstream of one or more RNA genes (RNA), TTSs adjacent to a centromere (Cent), and TTSs at telomeres (Telo). Panel G (top strand) is also shown in Fig. 5D. Download FIG S4, PDF file, 0.3 MB. Copyright © 2021 Jensen et al. 2021 Jensen et al. https://creativecommons.org/licenses/by/4.0/ This content is distributed under the terms of the Creative Commons Attribution 4.0 International license.

10.1128/mSphere.01204-20.5 FIG S5 Effect of depletion of JBP3 on transcription initiation. An analysis similar to the one in Fig. S4 in the supplemental material is shown, displaying the normalized read coverage for the 10-kb regions surrounding the transcription start sites. Plots are oriented such that transcription starts at position “0” and proceeds left to right, with the top strand being the coding strand to the right of the TSS. The normalized read counts for all TSSs mapping are shown. Panels on the left depict reads mapping to the top strand, and panels on the right depict reads mapping to the bottom strand. (A) All TSSs. (B) TSSs in dSSRs lacking either an RNA gene or a centromere. (C) Unidirectional (“head-to-tail”) TSSs. (D) TSSs immediately downstream of an RNA gene. (E) TSSs adjacent to a centromere. (F) TSSs adjacent to a telomere. (G) Box-and-whisker plots showing the median coverage in the 5-kb downstream of all TSSs (All), TSSs at cSSRs (Div), TSSs between head-to-tail PTUs (Uni), TSSs immediately downstream of one or more RNA genes (RNA), TSSs adjacent to a centromere (Cent), and TSSs at telomeres (Telo). Download FIG S5, PDF file, 0.4 MB. Copyright © 2021 Jensen et al. 2021 Jensen et al. https://creativecommons.org/licenses/by/4.0/ This content is distributed under the terms of the Creative Commons Attribution 4.0 International license.

## DISCUSSION

**FIG 6:**
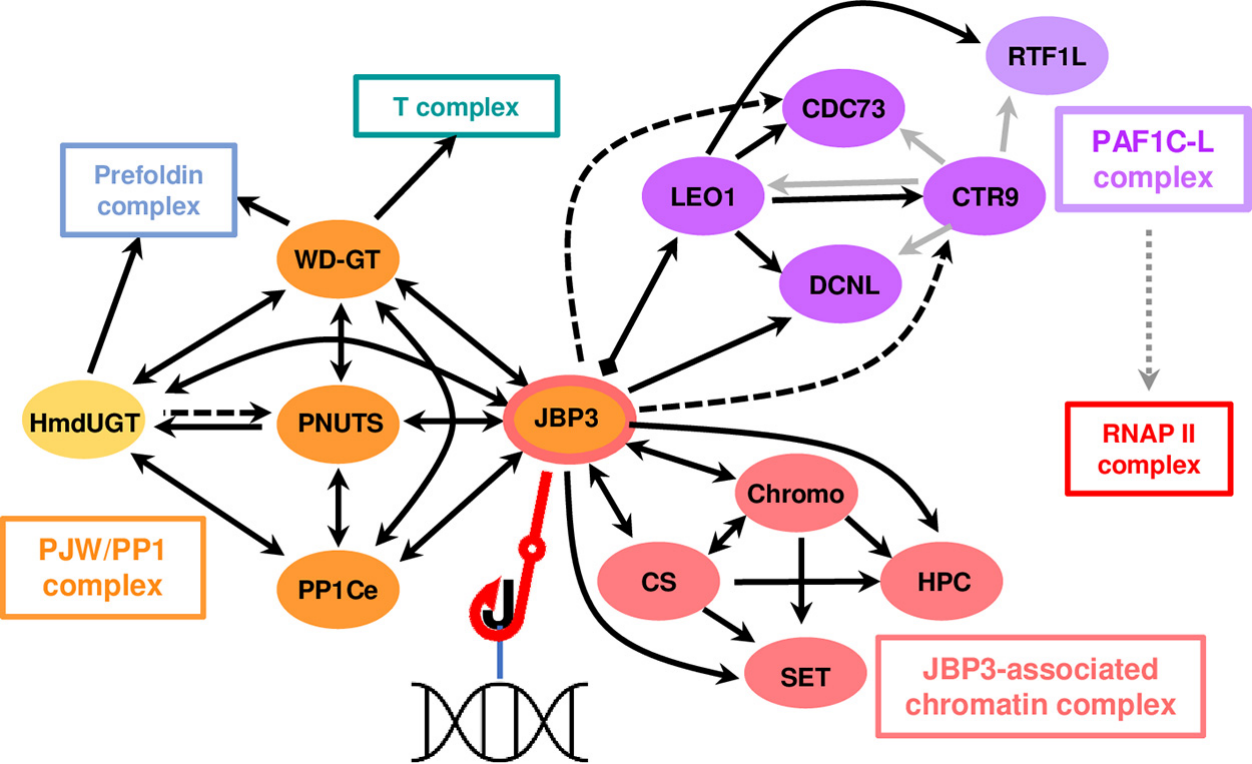
Network of interactions between JBP3-associated protein complexes. Solid lines denote proteins enriched in both replicates of the TAP-tag pulldowns, and dashed lines indicate proteins enriched in only one sample. Double-headed lines represent reciprocal enrichment with both proteins used as bait, while a diamond shape at one end of the line indicates that JBP3 was not detected in the LEO1 pulldown. Lines with a single arrowhead indicate that reciprocal enrichment was not attempted. Gray arrows represent interactions identified by copurification of proteins with CTR9 in T. brucei (38). Subunits within the three distinct JBP3-associated protein complexes are denoted by different colors, while the chaperone and RNA polymerase complexes are represented by boxes without individual components. The dotted line connecting the PAFC1-L complex to RNAP II reflects the interactions observed in other organisms. The interaction between JBP3 and base J is marked by the red fishhook.

Using the trypanosomatid-specific GT, which carries out the second step of J biosynthesis, as an entrée to search for the molecular machinery associated with the regulation of transcription in *Leishmania*, we have identified a network of three protein complexes that contain conserved building blocks often used to assemble molecular machinery regulating transcription in other eukaryotes. A novel J-binding protein (JBP3) lies at the nexus of these complexes (Fig. 6) and provides new insight into the molecular mechanism(s) used to mediate transcription termination at the end of the polycistronic transcription units emblematic of these (and related trypanosomatid) parasites. We have shown that JBP3 plays a central role in controlling the termination of RNAP II transcription since the depletion of JBP3 leads to defects in transcriptional termination at the 3′ ends of PTUs in *Leishmania* (Fig. 5), just as it does in T. brucei (20). However, readthrough transcription is not seen to the same extent at all TTSs. The presence of RNAP III-transcribed RNA genes downstream of the TTS appears to effectively block RNAP II, as we have seen previously for *jbp2* null mutants (11), and there is little or no readthrough at TTSs immediately upstream of centromeres and telomeres. This suggests that factors other than JBP3 may also play a role in reducing transcriptional readthrough at these loci. Alternatively, it is possible that the higher J content at centromeres and telomeres may more effectively “capture” what little JBP3 remains in the Tet^−^ cells. In contrast to the recent results from T. brucei (20), we find little evidence for antisense transcription at the 5′ ends of PTUs in *Leishmania*, even after the depletion of JBP3 levels. Divergent strand-switch regions (dSSRs), where adjacent PTUs are on opposite strands, tend to be smaller in *Leishmania* than those in T. brucei and lack the J-containing DNA found in the latter, suggesting that there may be little inappropriate antisense transcription in these regions. However, we find evidence that the depletion of JBP3 in *Leishmania* results in the upregulation of mRNA levels for protein-coding genes at the 3′ ends of PTUs (see Table S2 in the supplemental material). It is possible that this phenomenon is due to a more efficient polyadenylation of transcripts caused by the uncovering of cryptic *trans*-splicing sites downstream of the normal TTS. The toxic effects of a gradual accumulation of proteins from these mRNAs may be one explanation for the lag between the appearance of defects in transcription termination (day 2) and the decrease in the growth rate (day 4). This lag period also indicates that readthrough transcription is not an artifact of a reduced growth rate.

Our initial TAP-tagging experiments showed that GT associates (directly or indirectly) with four other proteins that resemble components of the PTW/PP1 complex, which plays a role in regulating transcriptional activity in other eukaryotes. The mammalian PTW/PP1 complex (containing PNUTS, TOX4, WDR82, and PP1C) has been implicated in numerous different cellular processes, including control of chromatin structure during cell cycle progression (27), repair of DNA damage by nonhomologous end joining (41), maintenance of telomere length (41), and developmental regulation of transcription (42). The *Leishmania* PJW/PP1 complex shows obvious parallels to the metazoan PTW/PP1 complex by incorporating analogous (although not necessarily homologous) proteins, with JBP3 replacing the DNA-binding function of TOX4. However, there are some interesting differences between *Leishmania* PJW/PP1 and the homologous T. brucei complex, most notably the absence of PP1C in the latter, where the complex was therefore called PJW (20). This absence is intriguing in the light of a recent publication that showed that the mammalian PTW/PP1 complex dephosphorylates the transcription elongation factor Spt5, thereby causing the RNAP II transcription complex to decelerate within the termination zone downstream of poly(A) sites and allowing the Xrn2 exonuclease to “track down and dislodge” the polymerase from the DNA template (30). This suggests that *Leishmania* and American trypanosomes may use PJW/PP1 to dephosphorylate Spt5 and mediate transcription termination but that African trypanosomes do not use this mechanism. In addition, although T. brucei encodes an orthologue of GT, it was not found to be associated with the PJW complex, pointing to another potential biological difference from *Leishmania* (or perhaps merely reflecting our use of a more rapid and sensitive purification protocol). In mammalian PTW/PP1C, PNUTS contains not only the phosphatase inhibitor motif but also a nuclear localization signal and provides a “scaffold” for recruiting the other proteins. However, *Leishmania* PNUTS is much smaller and lacks an obvious nuclear localization signal, so it is possible that other proteins in the complex provide these functions. For example, JBP3 contains a domain with structural similarity to the N-terminal TFIIS protein interaction domain found in mammalian PNUTS, and WD proteins can act as a scaffold in other complexes (43, 44).

JBP3 is also present in a complex (J3C) that contains proteins with domains suggesting a role in chromatin modification and/or remodeling. One protein (SET-J3C) contains a SET domain, which is often present in histone methyltransferases (HMTs) that modify lysine and arginine residues in histone proteins. HMTs usually also contain cysteine-rich pre-SET and post-SET domains that play a crucial role in substrate recognition and enzymatic activity by coordinating zinc ions. SET-J3C contains a Zn finger domain downstream of the SET domain (Fig. S3D) that may fulfill similar functions, suggesting that it is likely to have HMT activity. Two other proteins (Chromo-J3C and CS-J3C) contain Chromo and/or Chromo shadow domains typically involved in the recognition of the methylated lysine residues on histone tails and may be functional homologues of the metazoan heterochromatin protein 1 (HP1) and/or fission yeast Swi6, which contain similar domains and are involved in the repression of gene expression by heterochromatin (34). Thus, it is possible that J3C facilitates the repacking of chromatin at J-containing regions of the genome, rendering them heterochromatin-like and inaccessible to RNA polymerases. The function of the HPC-J3C subunit is unknown at this time, but the T. brucei orthologue (encoded by *Tb927.1.4250*) shows structural similarity to subunits from different protein complexes implicated in several processes, including histone modification and/or chromatin remodeling, and it localizes to the nucleus (45). This suggests that the primary function of HPC-J3C may be to serve as a scaffold for the recruitment and/or stabilization of the J3C complex.

JBP3 also associates (albeit transiently and/or indirectly) with a third protein complex (PAF1C-L), providing another potential connection between J and the regulation of transcription. PAF1C-L contains proteins with functional domains similar to those in yeast and mammalian PAF1C, which associates with the large subunit of RNAP II (35, 46) and plays a critical role in transcription elongation and termination (47, 48). Four proteins (PAF1, CDC73, LEO1, and CTR9) are consistently found as components of PAF1C in all other eukaryotes, while RTF1 (49) and the WD40-containing protein Ski8/WDR61 (50) show a less ubiquitous association. The parallels between the mammalian and trypanosomatid complexes are obvious since homologues of the CDC73, LEO1, and CTR9 subunits copurify with JBP3, and experiments performed by others in T. brucei showed that CTR9 has a tight association with LEO and CDC73 (38). Our TAP tag experiments using LEO1 as the bait protein showed enrichment of CDC73 and CTR9, along with a protein containing a Plus-3 domain similar to that in RTF1. Interestingly, the Plus-3 domain of RTF1 has been implicated in binding to Spt5 (51), which is tantalizing in light of the potential role for the PJW/PP1 complex in dephosphorylating this transcription factor (30). We (and others) failed to identify a convincing homologue of Ski8/WDR61 (or any other WD40 protein) in the PAF1C-L complex, but this protein is not tightly associated with mammalian PAF1C either. Remarkably, the namesake of the complex (PAF1) is absent from pulldowns of LEO and CTR9 in L. tarentolae and T. brucei, respectively, even though it (along with CTR9) is essential for the assembly of the complex in both yeast and humans (52). Moreover, extensive bioinformatic analysis of the trypanosomatid genomes failed to identify a homologue of PAF1, suggesting that it is truly absent from the *Leishmania* and *Trypanosoma* PAF1C-L complex. However, PAF1C-L contains an additional, trypanosomatid-specific component (DCNL) that has a putative protein-binding domain with structural similarity to the PONY/DCUN1 domain found in the eukaryotic DCN protein family. In other eukaryotes, DCN1 is required for the neddylation of cullin in SCF-type E3 ubiquitin ligase complexes that mark cellular proteins for proteasomal degradation (53). It is interesting to speculate that DCNL may be involved in PAF1C-L recruitment/function by interaction with N-terminal acetylated residues on histones and/or other chromatin-associated proteins. Whether DCNL functionally replaces PAF1 will remain an open question until its molecular function is dissected in more detail.

We have previously postulated that J might terminate transcription by directly preventing the progression of polymerase (11). The data presented here suggest three alternative, but not mutually exclusive, hypotheses based on the ability of JBP3 to bind base J. First, JBP3 may recruit the PJW/PP1 complex to J-containing regions, where it helps stall the elongating RNAP II by dephosphorylating Spt5 and causing the transcription complex to decelerate within the termination zone. Second, the J3C complex may enhance termination by modifying and/or remodeling the chromatin at J-containing regions, thereby preventing the passage of RNAP II. Third, we speculate that the interaction between JBP3 and PAF1C-L may also tether the RNAP II to the termination zone, where the DCNL subunit ubiquitinates the polymerase complex, promoting its degradation by the proteasome (54, 55). While disruption of the PJW complex in T. brucei (20) provides additional evidence for the first hypothesis, it remains to be seen whether disruption of J3C and PAF1C-L complexes also causes a defect in transcription termination.

While our findings provide several novel insights into the role of base J in transcription termination, they also raise several interesting questions. For example, why is GT part of the PJW/PP1 complex? One could envisage that the recruitment of the PJW/PP1 complex to regions of the genome containing J recruits may allow the more efficient glucosylation of nearby HmdU residues. Our proteomics data suggest that JBP3 interacts to various degrees with three different protein complexes (J3C, PJW/PP1, and PAF1C-L). It will be important to understand how these interactions are regulated. Are the complexes present at the same region simultaneously, or are they temporally and/or spatially segregated? For example, PJW/PP1 may be present at the ends of all PTUs, while J3C may be associated with only centromeres and/or telomeres. What histone modification/remodeling is mediated by J3C, and what role does this play in transcription termination? The availability of modern genome-wide approaches will no doubt provide the appropriate tools to answer these questions.

## MATERIALS AND METHODS

### Plasmid construction.

To create an expression vector that expresses epitope-tagged transgenes in *Leishmania*, the MHTAP tag (which bears a Myc epitope, six histidines, a protein A domain, and calmodulin-binding peptide [CBP]) was amplified from the plasmid pLEW-MHTAP (56) with the primers MHTAP-BamHI-S and MHTAP-NotI-AS (all primers used in this study are described in Table S3 in the supplemental material). Following cleavage with BamHI and NotI, the PCR fragment was inserted into BglII- and NotI-digested pLEXSY-I-bleCherry3 (Jena Biosciences). The resulting plasmid (pLEXSY-MHTAP; Fig. S6) allows the TAP tagging of the introduced coding regions under the control of a Tet-regulated T7 promoter and insertion into the *ODC* locus on chromosome 12 of L. tarentolae. We used a combination of previously reported data sets to identify the 5′ ends of L. tarentolae mRNAs as marked by the 39-bp splice leader sequence (11) and ribosome profiling data from Leishmania donovani (B. Jensen, R. Koren, G. Ramasamy, A. Haydock, A. Sekar, J. McDonald, D. Zilberstein, and P. J. Myler, unpublished data) to identify the correct coding DNA sequence (CDS) for bait proteins. CDSs were PCR amplified and digested with the restriction enzymes indicated in Table S3 prior to cloning.

10.1128/mSphere.01204-20.9 TABLE S3 Oligonucleotide primers used for construct creation. Primer names include the restriction sites (underlined bases) used for cloning into pLEXSY-MHTAP. Bases preceding the restriction site were added to facilitate restriction digestion of the PCR fragments. For the primers used for generating fragments for CRISPR/Cas9 deletion, the bases in boldface type match the sequence flanking the JBP3 gene. Download Table S3, PDF file, 0.1 MB. Copyright © 2021 Jensen et al. 2021 Jensen et al. https://creativecommons.org/licenses/by/4.0/ This content is distributed under the terms of the Creative Commons Attribution 4.0 International license.

10.1128/mSphere.01204-20.6 FIG S6 Map of plasmid pLEXSY-MHTAP showing key features and restriction sites. Bait proteins were cloned into the listed restriction sites immediately upstream of the TAP tag. Download FIG S6, PDF file, 0.06 MB. Copyright © 2021 Jensen et al. 2021 Jensen et al. https://creativecommons.org/licenses/by/4.0/ This content is distributed under the terms of the Creative Commons Attribution 4.0 International license.

### Parasite strains and tissue culture.

The Leishmania tarentolae Parrot-TarII wild-type (WT) and T7-TR strains (Jena Bioscience) were grown in SDM-79 medium supplemented with 10% fetal bovine serum. Strain T7-TR has constitutively expressed T7 RNA polymerase and Tet repressor (TetR) genes integrated into the ribosomal DNA (rDNA) locus, allowing for Tet-induced expression of integrated (or ectopically expressed) genes. Nourseothricin and hygromycin B were added to the medium at 100 μM to maintain the expression of T7 RNA polymerase and TetR.

### Tandem affinity purification of tagged protein complexes.

Ten micrograms of the SwaI-digested pLEXSY-MHTAP plasmid encoding a TAP-tagged protein was electroporated into the L. tarentolae WT and T7-TR cell lines as described previously (57), and transfectants were selected with 100 μg/ml bleomycin and maintained in 20 μg/ml bleomycin (plus nourseothricin and hygromycin B as described above). Proteins associated with the TAP-tagged “bait” were purified from 500 ml of cells following culture overnight (in the presence of 2 μg/ml tetracycline for T7-TR transfectants) as described previously (56), except that NP-40 was omitted from the final four washes of the proteins on the calmodulin beads and from the calmodulin elution buffer. The protocol went from lysis of cells to purified samples within 6 h. A sample from each pulldown (5% of the total eluate) was separated by 4 to 20% SDS-PAGE, and proteins were visualized using SilverQuest stain (Thermo Fisher Scientific, Life Technologies). Fractions containing a protein with the predicted molecular weight of the bait (usually fractions 2 and 3) were pooled. The same fractions were pooled from mock TAPs of the control parental line not expressing any bait protein.

### Western blotting.

Proteins from transfected cells were separated by SDS-PAGE on 4-to-20% gradient gels, transferred onto nitrocellulose, and detected with either mouse anti-6×His (Clontech) at 0.25 μg/ml or rabbit antibody against calmodulin-binding peptide (GenScript) at 0.1 μg/ml, with rabbit antibody against T. brucei phosphoglycerate kinase serving as a control (58). Primary antibodies were detected with goat anti-rabbit Ig conjugated with Alexa Fluor 680 (50 ng/ml) or goat anti-mouse Ig conjugated with IRDye 800 (25 ng/ml) and imaged on the Li-Cor Odyssey CLX system.

### Proteomic analysis.

Pooled protein fractions were denatured with 6 M urea, reduced with 5 mM dithiothreitol, alkylated with 25 mM iodoacetamide, and digested at 37°C for 3 h using 1:200 (wt/wt) endoproteinase Lys-C (Thermo Fisher Scientific). The urea was then diluted to 1.5 M, and samples were further digested at 37°C overnight with 1:25 (wt/wt) trypsin (Thermo Fisher Scientific). Proteinase activity was stopped with formic acid, and peptides were purified using C_18_ reversed-phase chromatography (Waters), followed by hydrophilic interaction chromatography (HILIC; Nest Group). Purified peptides were separated by online nanoscale high-performance liquid chromatography (HPLC) (Easy-nLC II; Proxeon) with a C_18_ reversed-phase column (Magic C_18_ AQ 5 μm, 100 Å) over an increasing 90-min gradient of 5 to 35% buffer B (100% acetonitrile, 0.1% formic acid) at a flow rate of 300 nl/min. Eluted peptides were analyzed with an Orbitrap Elite mass spectrometer (Thermo Fisher Scientific) operated in data-dependent mode, with the 15 most intense ions per MS1 survey scan selected for MS2 fragmentation by rapid collision-induced dissociation (rCID) (59). MS1 survey scans were performed in the Orbitrap instrument at a resolution of 240,000 at *m/z* 400 with charge state rejection enabled, while rCID MS2 was performed in the dual linear ion trap with a minimum signal of 1,000. Dynamic exclusion was set to 15 s.

Raw output data files were analyzed using MaxQuant (v1.5.3.30) (60) to search against a proteome predicted after resequencing and annotation of the L. tarentolae Parrot (LtaP) genome (A. Sur, J. McDonald, G. Ramasamy, and P. J. Myler, unpublished data). A reverse sequence decoy database was used to impose a strict 1% false discovery rate (FDR) cutoff. Label-free quantification was performed using the MaxLFQ algorithm (61), and further data processing was performed in Perseus (v1.5.3.1) (62) and Microsoft Excel. To avoid zero-value denominators, null values in the remaining data were replaced by imputation using the background signal within one experiment using Perseus. Nonparasite contaminants, decoys, and single-peptide identifications among all samples in an experiment were removed. Proteins were deemed to be part of a complex associated with the bait protein if at least two peptides were detected and the protein showed >32-fold (log_2_ fold change of >5) enrichment (compared to the control not expressing the bait) in both replicates. In a few cases, proteins showing >100-fold enrichment in a single replicate only were also considered potential subunits of the complex. Proteins with 1,024-fold (log_2_ fold change of <10) less enrichment than the bait protein were assumed to be copurifying contaminants and (usually) ignored.

### Bioinformatic analysis of protein function.

Structure-based similarity searches for known domains were performed with HHpred (23). Domain boundaries for the JBP3 DNA-binding domain (DBD) were refined by aligning trypanosomatid sequences that clustered in the same OrthoMCL group as JBP3 with T-Coffee (63). Homology models of the JBP3 DBD domain using RosettaCM (64) were built with the L. tarentolae JBP1 structure (PDB accession number 2XSE) as a template. The top-scoring model covered residues 111 to 312 of the JBP3 DBD with a confidence score of 0.67.

### Deletion of JBP3 using SaCas9.

JBP3 was deleted using Staphylococcus aureus Cas9 (SaCas9)-directed cleavage of sites flanking the endogenous locus as described previously (40). Briefly, guide RNAs (gRNAs) directed at sites for SaCas9 cleavage were generated *in vitro* using T7 Megashort from Thermo Fisher from PCR-generated templates. The 5′ and 3′ gRNA sequences used were GATGTGAAACGCTAAGCAGTCCCGAGT and AGGAACGAAAGCACACAGCAGAGGAGT, where the protospacer-adjacent motif (PAM) sites are underlined. Repair fragments containing a drug resistance gene were generated as described previously (65) from pTNeo or pTPuro templates using primers LtJBP3-up and LtJBP3-down. Heat-denatured guide RNA was complexed with 20 μg SaCas9 recombinant protein at an equimolar ratio and incubated for 15 min at room temperature before mixing with 2 μg of each repair fragment that had been ethanol precipitated and resuspend in Tb-BSF (*T. brucei* bloodstream form) buffer (66). L. tarentolae T7-TR cells were grown overnight (in the presence of 2 μg/ml tetracycline for the latter), pelleted, washed with phosphate-buffered saline (PBS), and resuspended in 100 μl Tb-BSF buffer. For each transfection, 10^6^ cells were mixed with the SaCas9/guide RNA complexes and repair fragments and electroporated in an Amaxa Nucleofector using program X-001. Immediately following transfection, cells were split into three flasks. After allowing the cells to recover overnight, antibiotics were added to the flasks, one of which was grown in 10 μg/ml G418, one of which was grown in 4 μg/ml puromycin, and one of which was grown with both drugs. Deletion of the endogenous *JBP3* gene(s) was confirmed by PCR amplification of genomic DNA using primers JBP3-M84P and JBP3-P2147M that flank the region being deleted. Clone cell lines were obtained by limiting dilution of the transfectants, and clones were retested for JBP3 deletion by PCR. While we were able to obtain clones where both endogenous copies of *JBP3* were deleted by selection with either puromycin or neomycin, we were unable to obtain lines where both drugs were used.

### RNA-seq analysis.

RNA was isolated using TRIzol (Thermo Fisher Scientific) and resuspended in 10 mM Tris (pH 7), and RNA quality was assessed using the Bioanalyzer 6000 Pico chip (Agilent). mRNA was isolated from 1 μg total RNA using the New England BioLabs (NEB) poly(A) mRNA magnetic isolation module and prepared using the stranded RNA-seq protocol (67), modified for *Leishmania* as described previously (68). Libraries were sequenced on an Illumina HiSeq instrument, obtaining paired-end 150-bp reads. Reads were aligned against our in-house L. tarentolae genome with Bowtie2 (69) using the “very high sensitivity” parameter or the Geneious assembler (Geneious Prime 11.05) using the “low sensitivity/fastest” option. Differential expression analysis was performed on the Geneious assemblies using the DESeq2 module to compare Tet^+^ and Tet^−^ samples from days 2, 3, 4, 6, and 8 for replicate 1 and from days 2, 3, 4, 5, 7, and 9 for replicate 2. Strand-specific read coverage was calculated directly from BAM files of the Bowtie2 alignments using customized pysam scripts (https://github.com/pysam-developers/pysam).

### Data availability.

Mass spectrometry data were deposited in the MassIVE database (https://massive.ucsd.edu/ProteoSAFe/static/massive.jsp) and can be accessed from ProteomeXchange under accession number PXD020779. RNA-seq data were deposited in the Sequence Read Archive (SRA) at the NCBI and can be accessed under BioProject accession number PRJNA657890.
