# Supplementary figures and images for: Chromatin-Associated Protein Complexes Link DNA Base J and Transcription Termination in Leishmania
Source: mSphere. 2021 Feb 24;6(1):e01204-20. doi: 10.1128/mSphere.01204-20 (PMC8544896; doi:10.1128/mSphere.01204-20)

Figure S1

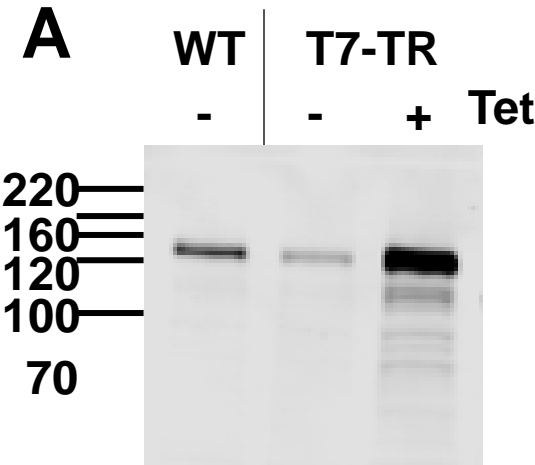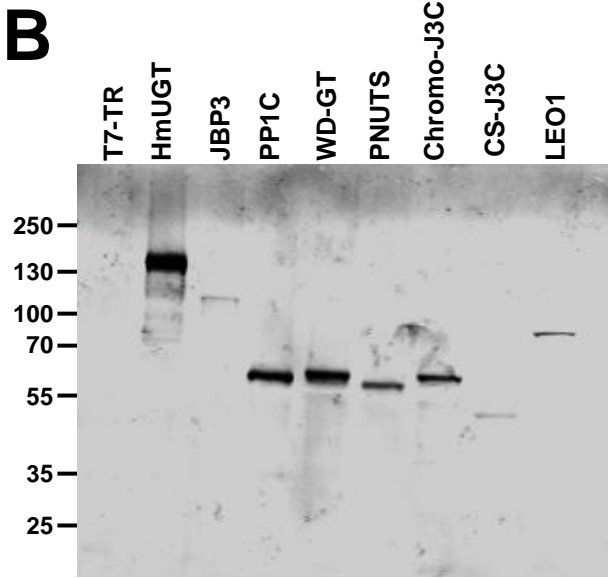

Supplement: FIG S1 [file msphere.01204-20-sf001.pdf]

Figure S4

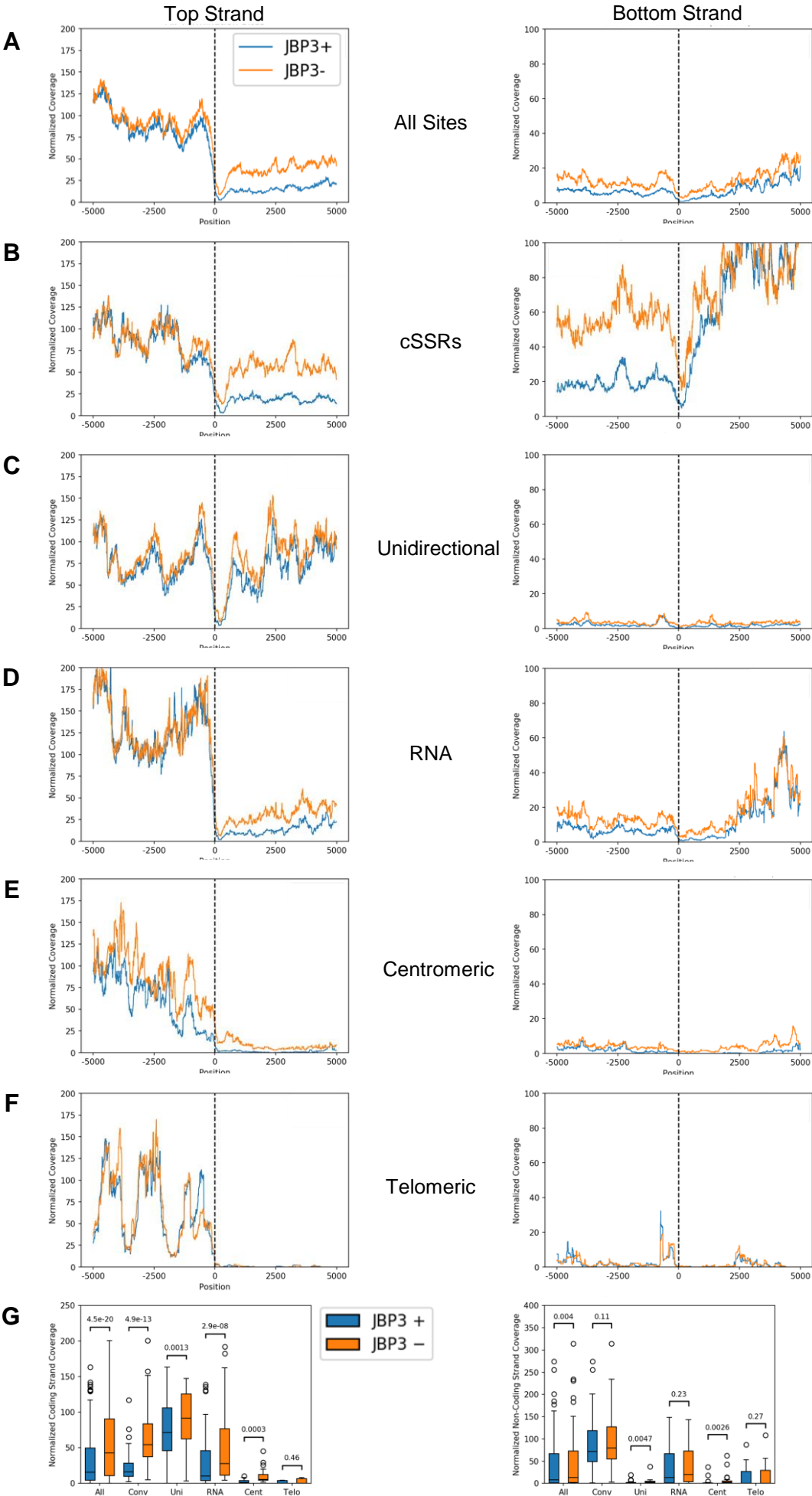

Supplement: FIG S4 [file msphere.01204-20-sf004.pdf]

Figure S5

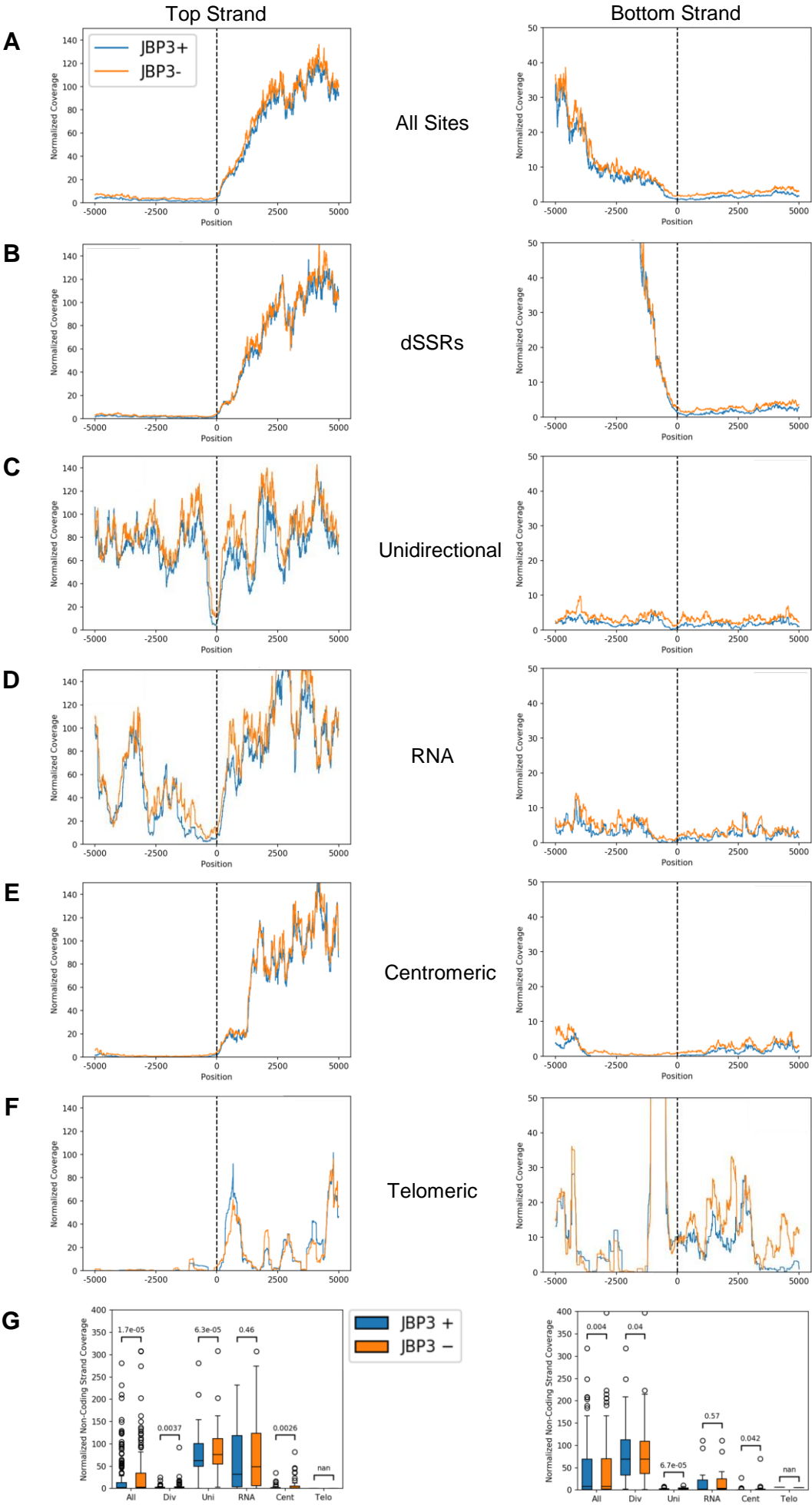

Supplement: FIG S5 [file msphere.01204-20-sf005.pdf]

Figure S6

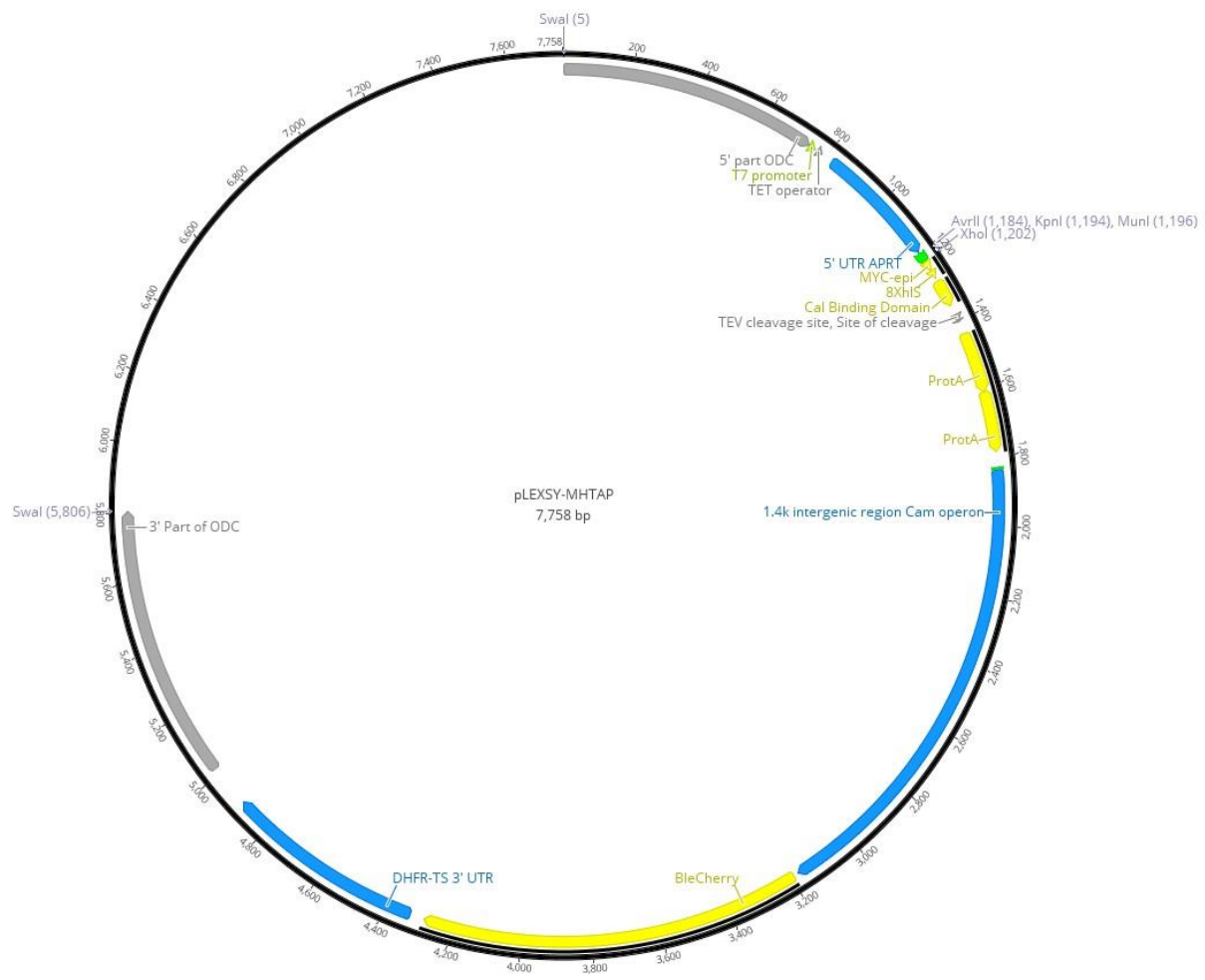

Supplement: FIG S6 [file msphere.01204-20-sf006.pdf]
