# Supplementary material for: Chromatin-Associated Protein Complexes Link DNA Base J and Transcription Termination in Leishmania
Source: mSphere. 2021 Feb 24;6(1):e01204-20. doi: 10.1128/mSphere.01204-20 (PMC8544896; doi:10.1128/mSphere.01204-20)
Supplement: TABLE S1 [file msphere.01204-20-st001.pdf]

Table S1. Protein complexes identified by TAP-tagged experiments

Protein name		MW	TriTrypDB annotation (Gene ID)	# of peptides		log_2 fold-enrichment		
Bait	Pull-down	(kDa)		Rep 1	Rep 2	Rep 1	Rep 2	Avg
HmdUGT	HmdUGT	101.3	Hypothetical protein, conserved (LtaP36.2450)	55	41	13.7	14.6	14.1
	JBP3	73.9	Hypothetical protein, conserved (LtaP36.0380)	19	7	13.6	8.6	11.1
	WD-GT	38.6	Hypothetical protein, conserved (LtaP32.3990)	13	7	11.7	6.9	9.3
	PP1Ce	42.3	Protein phosphatase 1 catalytic subunit, putative (LtaP15.0230)	10	3	9.8	5.1	7.4
	PNUTS	28.6	Hypothetical protein, conserved (LtaP33.1440)	8	0	9.8	-	4.9
	PFDN3	22.3	Prefoldin-like protein (LtaP26.1310)	6	4	8.8	7.8	8.3
	PFDN6	15.8	Prefoldin subunit, putative (LtaP05.1280)	4	3	8.0	6.0	7.0
	PFDN5	18.0	Prefoldin 5-like protein (LtaP22.0650)	3	2	8.4	5.5	6.9
	PFDN4	14.9	Hypothetical protein, conserved (LtaP25.0870)	2	2	7.0	5.7	6.4
	PFDN1	16.5	Hypothetical protein, conserved (LtaP30.0960)	4	2	6.3	3.7	5.0
	PFDN2	15.3	Prefoldin subunit 2, putative (LtaP09.0680)	1	0	5.6	-	2.8
PP1Ce	PP1Ce	42.3	Protein phosphatase 1 catalytic subunit, putative (LtaP15.0230)	16	21	17.1	17.1	17.1
	WD-GT	38.6	Hypothetical protein, conserved (LtaP32.3990)	21	25	12.6	17.1	14.8
	PNUTS	28.6	Hypothetical protein, conserved (LtaP33.1440)	15	15	12.9	12.1	12.5
	JBP3	73.9	Hypothetical protein, conserved (LtaP36.0380)	28	34	11.8	12.8	12.3
	HmdUGT	101.3	Hypothetical protein, conserved (LtaP36.2450)	23	25	4.8	9.6	7.2
	PPP1R7	44.7	Protein phosphatase type 1 regulator-like protein (LtaP05.1290)	26	35	15.8	15.0	15.4
	PPP1R11	16.5	Protein phosphatase inhibitor, putative (LtaP07.0770)	11	7	14.3	13.6	13.9
	PPP1R2	15.5	Protein phosphatase inhibitor 2, IPP-2, putative (LtaP29.0170)	8	8	11.7	12.9	12.3
	DNAJ	43.7	Heat shock protein DNAJ, putative (LtaP27.2520)	6	9	7.7	5.6	6.6
PNUTS	PNUTS	28.6	Hypothetical protein, conserved (LtaP33.1440)	16	17	17.1	18.2	17.7
	WD-GT	38.6	Hypothetical protein, conserved (LtaP32.3990)	22	22	12.2	13.2	12.7
	PP1Ce	42.3	Protein phosphatase 1 catalytic subunit, putative (LtaP15.0230)	16	15	12.1	12.3	12.2
	JBP3	73.9	Hypothetical protein, conserved (LtaP36.0380)	30	24	10.0	10.6	10.3
	HmdUGT	101.3	Hypothetical protein, conserved (LtaP36.2450)	14	14	9.4	7.0	8.2
WD-GT	WD-GT	38.6	Hypothetical protein, conserved (LtaP32.3990)	21	25	13.9	18.6	16.3
	PP1Ce	42.3	Protein phosphatase 1 catalytic subunit, putative (LtaP15.0230)	15	17	13.3	14.6	13.9
	PNUTS	28.6	Hypothetical protein, conserved (LtaP33.1440)	14	15	13.5	13.0	13.2
	JBP3	73.9	Hypothetical protein, conserved (LtaP36.0380)	32	36	11.6	13.5	12.5
	HmdUGT	101.3	Hypothetical protein, conserved (LtaP36.2450)	26	23	8.1	10.3	9.2
	PFDN6	15.8	Prefoldin subunit, putative (LtaP05.1280)	8	10	10.8	9.5	10.2
	PFDN2	15.3	Prefoldin subunit 2, putative (LtaP09.0680)	3	4	10.2	6.7	8.4
	PFDN5	18.0	Prefoldin 5-like protein (LtaP22.0650)	3	5	9.4	8.4	8.9
	PFDN4	14.9	Prefoldin subunit, putative (LtaP25.0870)	3	3	8.8	8.8	8.8
	PFDN1	16.5	Prefoldin subunit, putative (LtaP30.0960)	7	3	8.8	5.5	7.1
	PFDN3	22.3	Prefoldin-like protein (LtaP26.1310)	6	10	7.2	10.0	8.6
	CCT7	61.7	T-complex protein 1, eta subunit, putative (LtaP35.3870)	24	28	12.0	5.8	8.9
	CCT5	94.4	Chaperonin containing t-complex protein, putative (LtaP32.1090)	19	18	11.2	4.9	8.1
	CCT8	58.3	TCP-1/cpn60 chaperonin family, putative (LtaP36.7170)	26	18	10.3	7.7	9.0
	CCT3	60.1	T-complex protein 1, gamma subunit, putative (LtaP23.1490)	20	21	10.1	8.3	9.2
	CCT1	59.2	Chaperonin alpha subunit, putative (LtaP32.3480)	9	11	7.3	6.7	7.0
	CCT4	59.6	T-complex protein 1, delta subunit, putative (LtaP21.1260)	18	17	7.0	5.5	6.2
	DNAJ	43.7	Heat shock protein DNAJ, putative (LtaP27.2520)	8	9	9.0	5.6	7.3
	HSP110	91.1	Heat shock protein 110, putative (LtaP18.1330)	8	15	6.8	7.5	7.1
	ZFK	144.0	Zinc finger protein kinase-like (LtaP28.1730)	10	6	8.0	5.4	6.7
	-	161.3	Hypothetical protein, conserved (LtaP33.2370)	14	12	7.7	6.1	6.9
JBP3	JBP3	73.9	Hypothetical protein, conserved (LtaP36.0380)	38	44	14.9	17.0	15.9
	SET-J3C	88.8	SET domain containing protein, putative (LtaP35.2400)	18	22	12.6	13.0	12.8
	CS-J3C	24.1	Hypothetical protein, conserved (LtaP28.2640)	10	8	13.5	11.5	12.5
	HPC-J3C[c]	93.5	Hypothetical protein, conserved (LtaP12.0900)	26	31	11.8	13.1	12.4
	Chromo-J3C	40.2	Chromo domain-containing protein (LtaP14.0150)	12	18	12.6	10.8	11.7
	WD-GT	38.6	Hypothetical protein, conserved (LtaP32.3990)	14	24	8.0	16.6	12.3
	PP1Ce	42.3	Protein phosphatase 1 catalytic subunit, putative (LtaP15.0230)	13	16	8.8	12.9	10.9
	PNUTS	28.6	Hypothetical protein, conserved (LtaP33.1440)	11	15	9.0	11.6	10.3
	HmdUGT	101.3	Hypothetical protein, conserved (LtaP36.2450)	30	27	9.6	11.6	10.6
	LEO1	63.2	RNA polymerase-associated protein, putative (LtaP35.2870)	11	12	8.1	6.8	7.4
	DCNL	68.6	Hypothetical protein, conserved (LtaP29.1270)	6	2	6.4	4.5	5.4
	CTR9	97.1	RNA polymerase-associated protein CTR9, putative (LtaP29.2750)	2	0	6.2	-	3.1
	CDC73	41.0	RNA polymerase-associated protein CDC73, putative (LtaP36.4090)	5	1	5.7	-0.5	2.6
Chromo-J3C	Chromo-J3C	40.2	Chromo domain containing protein (LtaP14.0150)	26	26	11.6	11.6	11.6
	HPC-J3C[c]	93.5	Hypothetical protein, conserved (LtaP12.0900)	26	26	10.4	9.2	9.8
	CS-J3C	24.1	Hypothetical protein, conserved (LtaP28.2640)	11	13	10.0	9.5	9.8
	SET-J3C	88.8	SET domain containing protein, putative (LtaP35.2400)	20	19	8.8	7.5	8.2
	JBP3	73.9	Hypothetical protein, conserved (LtaP36.0380)	26	25	8.6	7.1	7.9
CS-J3C	CS-J3C	24.1	Hypothetical protein, conserved (LtaP28.2640)	11	13	11.8	11.9	11.8
	SET-J3C	88.8	SET domain containing protein, putative (LtaP35.2400)	21	20	9.6	9.3	9.4
	Chromo-J3C	40.2	Chromo domain containing protein (LtaP14.0150)	23	21	9.1	8.5	8.8
	JBP3	73.9	Hypothetical protein, conserved (LtaP36.0380)	20	17	8.0	7.5	7.8
	HPC-J3C[c]	93.5	Hypothetical protein, conserved (LtaP12.0900)	29	27	6.2	6.4	6.3
	-	21.1	i/6 autoantigen-like protein (LtaP22.1440)	4	6	4.6	7.8	6.2
LEO1	LEO1	63.2	RNA polymerase-associated protein, putative (LtaP35.2870)	33	34	17.0	16.6	16.8
	DCNL	68.6	Hypothetical protein, conserved (LtaP29.1270)	35	35	14.0	13.8	13.9
	CDC73	41.0	RNA polymerase-associated protein (CDC73), putative (LtaP36.4090)	11	13	10.3	10.4	10.4
	CTR9	97.1	RNA polymerase-associated protein (CTR9), putative (LtaP29.2750)	28	30	9.6	9.3	9.5
	RTF1L	73.5	Hypothetical protein, conserved (LtaP14.0860)	18	19	4.2	3.5	3.8
	PEX12	50.8	Hypothetical protein, conserved (LtaP13.1250)	5	5	6.2	4.6	5.4
	TFIIS2-1	50.9	Transcription elongation factor-like protein (LtaP33.3050)	4	6	5.2	5.1	5.1
