# Supplementary material for: Chromatin-Associated Protein Complexes Link DNA Base J and Transcription Termination in Leishmania
Source: mSphere. 2021 Feb 24;6(1):e01204-20. doi: 10.1128/mSphere.01204-20 (PMC8544896; doi:10.1128/mSphere.01204-20)
Supplement: FIG S2 [file msphere.01204-20-sf002.pdf]

Figure S2

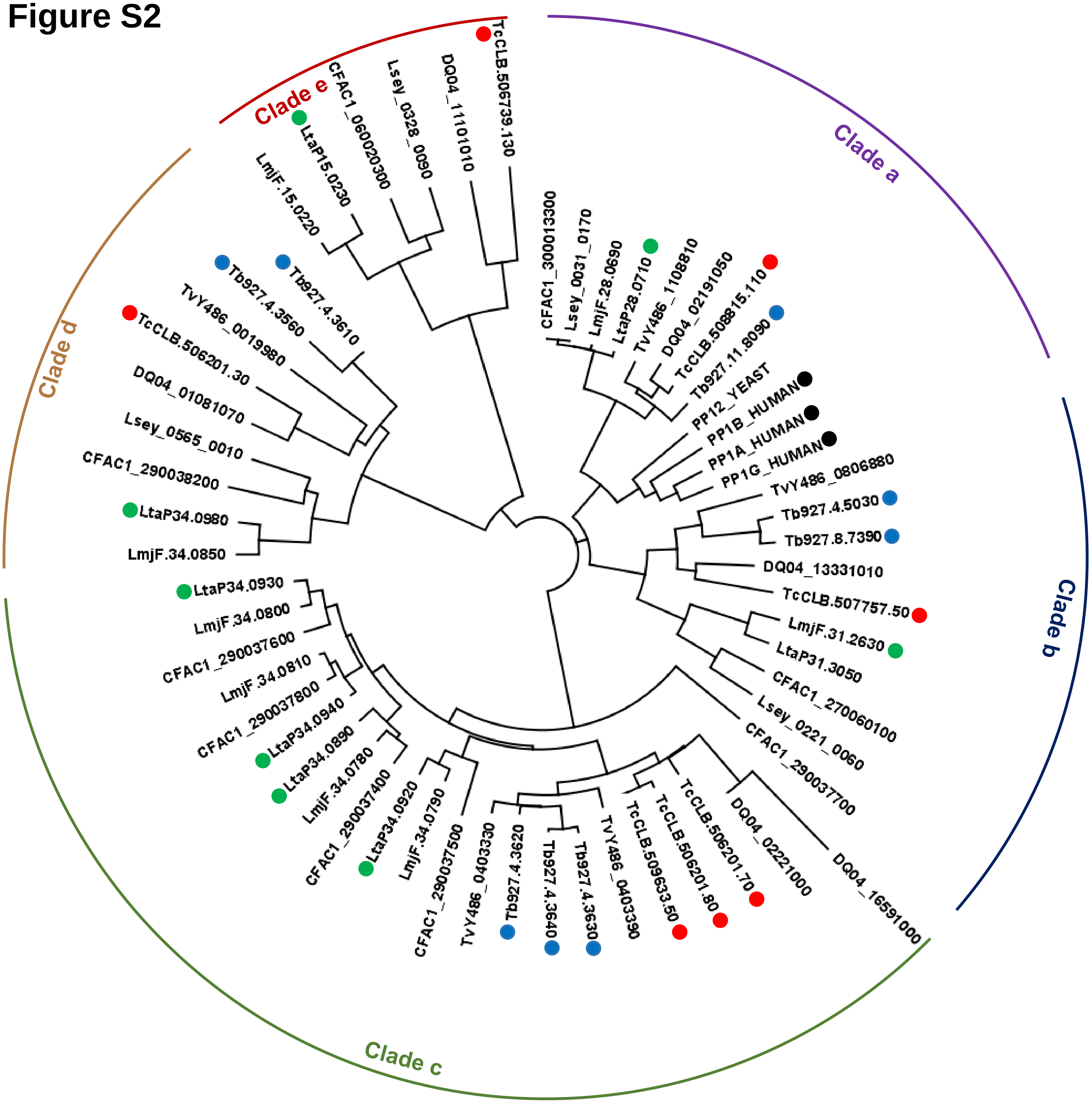

Leishmaniiae	*Crithidia fasiculata* (CFAC)<br>*Leishmania major* (LmjF)<br>*Leptomonas seymouri* (Lsey)<br>*Leishmani tarentolae* (LtaP) ●
Stercorarian trypanosomes	*Trypanosoma cruzi* (TcCLB) ●<br>*Trypanosoma grayi* (DQ04)
Salivarian trypanosomes	*Trypanosoma brucei* (Tb927) ●<br>*Trypanosoma vivax* (TvY)
	Homo sapiens (Human) ●<br>*Saccharomyces cerevisiae* (Yeast)
