# Supplementary material for: Chromatin-Associated Protein Complexes Link DNA Base J and Transcription Termination in Leishmania
Source: mSphere. 2021 Feb 24;6(1):e01204-20. doi: 10.1128/mSphere.01204-20 (PMC8544896; doi:10.1128/mSphere.01204-20)
Supplement: FIG S3 [file msphere.01204-20-sf003.pdf]

Figure S3A

PNUTS

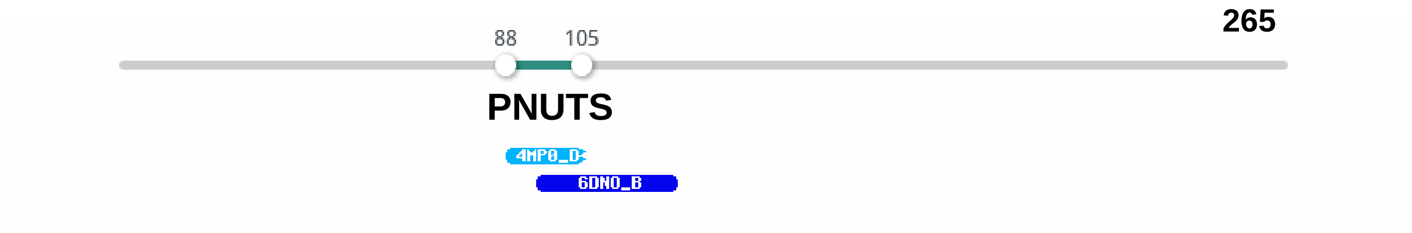

PNUTS

Hit	Name	Probability	E-value	SS	Cols	Target Length
4MP0_D	Serine/threonine-protein phosphatase PP1-alpha catalytic subunit; Serine/threonine phosphatase, Nucleus, HYDROLASE; HET: GOL, PO4; 2.1003A {Homo sapiens}; Related PDB entries: 4MOY_B 4MP0_B	55.07	9.2	1.6	18	44
6DNO_B	Serine/threonine-protein phosphatase PP1-alpha catalytic subunit; Inhibitor, complex, HYDROLASE, HYDROLASE-HYDROLASE INHIBITOR; HET: 1ZN; 1.45A {Homo sapiens}	38.83	30	2	30	30

Figure S3B

WD-GT

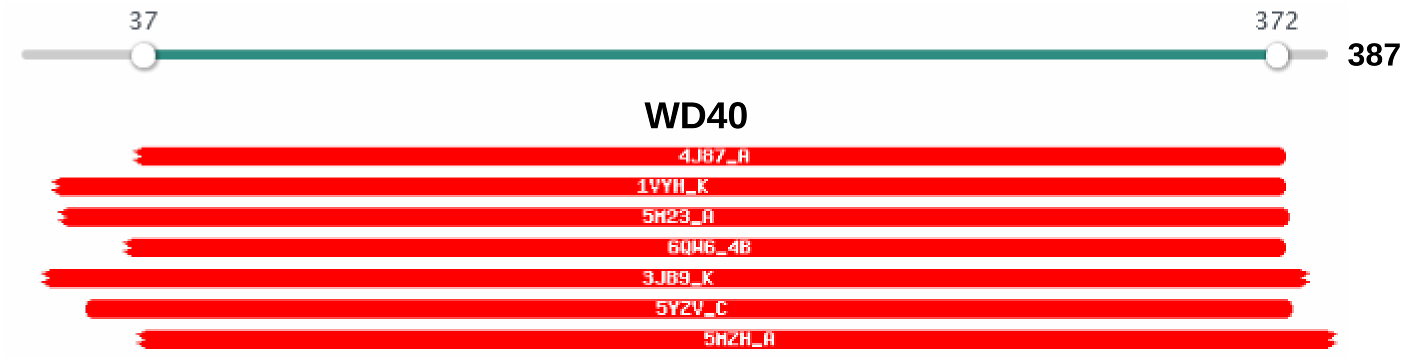

WD40

Hit	Name	Probability	E-value	SS	Cols	Target Length
4J87_A	coatomer subunit alpha; Beta propeller domain, Vesicle trafficking; 1.67A {Schizosaccharomyces pombe}; Related PDB entries: 6PBG_A 4J8G_B 4J8G_A 4J8B_A	100	1.1e-33	36.5	285	327
1VYH_K	PLATELET-ACTIVATING FACTOR ACETYLHYDROLASE IB BETA; LISSENCEPHALY, ACETYLHYDROLASE, PLATELET ACTIVACTING FACTOR; 3.4A {HOMO SAPIENS} SCOP: b.69.4.1; Related PDB entries: 1VYH_D 1VYH_C 1VYH_T 1VYH_P 1VYH_L 1VYH_H 1VYH_S 1VYH_O 1VYH_G	100	3.5e-33	40.5	307	410
5M23_A	WD repeat-containing protein 5; MLL1 Methyltransferase peptide complex WDR5; HET: 7DC; 1.97A {Homo sapiens} SCOP: b.69.4.0; Related PDB entries: 5M25_A	100	1.8e-33	37.9	310	434
6QW6_4B	Small nuclear ribonucleoprotein Sm D1; RNP complex, splicing, RNA, protein; HET: M7M, GTP, IHP; 2.92A {Homo sapiens}; Related PDB entries: 6AH0_K 6QX9_4B 6AHD_K 5O9Z_F 3JCR_L	100	6.3e-33	39.6	286	522
3JB9_K	Pre-mRNA-splicing factor spp42, Pre-mRNA-splicing factor; Spliceosome, U2/U5/U6, Lariat, RNA BINDING; HET: ADP, GDP; 3.6A {Schizosaccharomyces pombe 972h-} SCOP: b.69.4.1	100	2.6e-32	42.4	313	473
5YZV_C	Probable serine/threonine-protein kinase PkwA (E.C.2.7.11.1); prokaryotic, thermostable, reversible folding, PROTEIN; 2.6A {Thermomonospora curvata}; Related PDB entries: 5YZV_B 5YZV_E 5YZV_D 5YZV_A	100	2e-32	39	299	302
5MZH_A	Dynein assembly factor with WDR; Intraflagellar transport, cilium, beta propeller; HET: SO4; 2.398A {Chlamydomonas reinhardtii}; Related PDB entries: 5MZH_B	100	1.3e-32	39.8	296	452

Figure S3C

JBP3

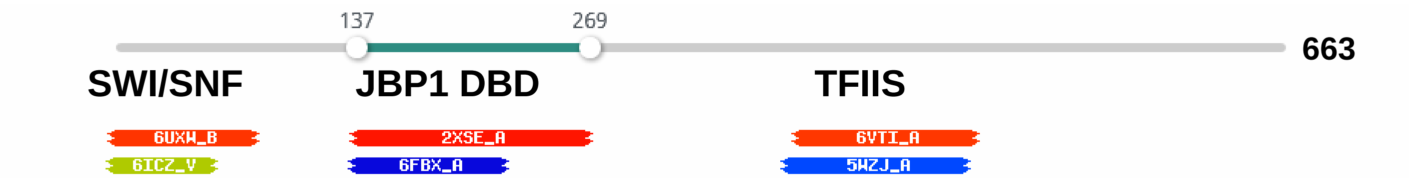

SWI/SNF

6UXW_B	Histone H3.2, Histone H4, Histone; SWI/SNF, chromatin remodeler, TRANSCRIPTION, TRANSCRIPTION-DNA; HET: ADP, PO4;{Saccharomyces cerevisiae (strain ATCC 204508 / S288c)}; Related PDB entries: 6UXV_B	95.6	0.011	4.1	82	1314
6ICZ_V	Protein mago nashi homolog 2; Human Post-catalytic Spliceosome, SPLICING; HET: SEP, ATP, GTP, I6P; 3.0A {Homo sapiens}; Related PDB entries: 6QDV_H 6FF7_T 5Z57_V 5XJC_V 5MQF_T 5Z56_V 5Z58_V 5YZG_V	80.3	1.2	2.6	60	908

JBP1 DBD

Hit	Name	Probability	E-value	SS	Cols	Target Length
2XSE_A	THYMINE DIOXYGENASE JBP1 (E.C.1.14.11.6); OXIDOREDUCTASE, DNA-BINDING; HET: MSE; 1.9A {LEISHMANIA TARENTOLAE}	98.2	0.000011	10.5	122	170
6FBX_A	BCL2-like 10, BCL2-antagonist of cell; Bcl-2 family, pro-survival protein, APOPTOSIS; 1.639A {Danio rerio}; Related PDB entries: 6H1N_A	37.57	210	7.9	81	152

TFIIS

6VTI_A	E3 ubiquitin-protein ligase UHRF1 (E.C.2.3.2.27); PP1-PNUTS phosphatase complex, Regulatory Subunit; NMR {Rattus norvegicus}	95.26	0.13	8.7	98	148
5WZJ_A	Pumilio homolog 23, RNA; RNA binding protein, SIGNALING PROTEIN; 2.101A {Arabidopsis thaliana}; Related PDB entries: 5WZK_A 5WZH_A 5WZI_A 5WZG_A	46.13	180	10.3	96	582

Figure S3D

SET-J3C

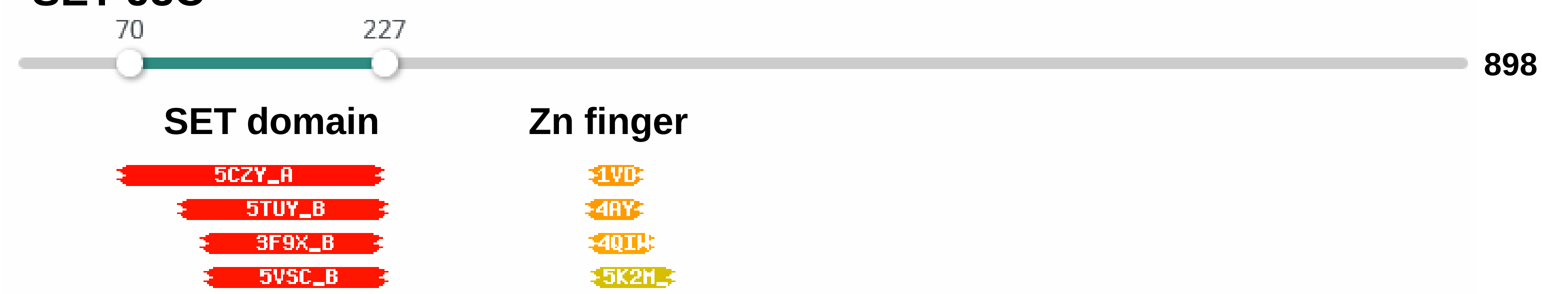

SET domain

Hit	Name	Probability	E-value	SS	Cols	Length
5CZY_A	Eukaryotic huntingtin interacting protein B; SET domain, TRANSFERASE; HET: SAM, GOL; 2.2A {Legionella pneumophila subsp. pneumophila str. Philadelphia 1}	98.55	1.8e-8	0.8	134	483
5TUY_B	Histone-lysine N-methyltransferase EHMT2 (E.C.2.1.1.-,2.1.1.43); Methyl-TRANSFERASE INHIBITOR complex, TRANSFERASE-TRANSFERASE INHIBITOR; HET: SAM, 7L6; 2.6A {Homo sapiens} SCOP: b.85.7.0; Related PDB entries: 5TUY_A	98.13	0.0000016	3.3	115	268
3F9X_B	Histone-lysine N-methyltransferase SETD8 (E.C.2.1.1.43), Histone; methyltransferase, histone, SET, lysine, Alternative; HET: MLY, SAH; 1.25A {Homo sapiens}; Related PDB entries: 1ZKK_B 1ZKK_A 1ZKK_D 1ZKK_C 3F9W_B 3F9W_A 3F9W_D 3F9W_C 3F9X_A 3F9X_D 3F9X_C 3F9Y_B 3F9Y_A 5W1Y_B	98.12	0.0000018	2.9	101	166
5VSC_B	Histone-lysine N-methyltransferase EHMT2 (E.C.2.1.1.-,2.1.1.43); protein-small molecule inhibitor complex, TRANSFERASE-TRANSFERASE; HET: SAM, 9HJ; 1.4A {Homo sapiens} SCOP: b.85.7.0; Related PDB entries: 3HNA_B 3HNA_A 5TTG_B 5TTG_A 5V9J_B 5V9J_A 4I51_B 4I51_A 3SW9_B	98.11	0.0000011	1.7	105	275

Zn finger

1VD4_A	Transcription initiation factor IIE, alpha; ZINC FINGER, Transcription; HET: ZN; NMR {Homo sapiens} SCOP: g.41.3.1	87.21	0.43	1.9	27	62
4AYB_P	DNA-DIRECTED RNA POLYMERASE (E.C.2.7.7.6); TRANSFERASE, MULTI-SUBUNIT, TRANSCRIPTION; HET: ZN; 3.202A {SULFOLOBUS SHIBATAE}; Related PDB entries: 3HKZ_X 2Y0S_P 4V8S_BP 2WB1_X 4V8S_AX 2WAQ_P 3HKZ_P 2PMZ_P 2Y0S_X 2PMZ_Z 2WB1_P	86.97	0.29	0.7	31	48
4QIW_W	DNA-directed RNA polymerase (E.C.2.7.7.6), DNA-directed; Transcription, DNA-directed RNA polymerase; HET: ZN; 3.5A {Thermococcus kodakarensis}; Related PDB entries: 4QIW_P	86.25	0.6	2	34	49
5K2M_K	RimK-related lysine biosynthesis protein, Probable; ATP-dependent amine/thiol ligase family Amino-group; HET: SO4, ADP, PO4, UN1; 2.18A {Thermococcus kodakarensis (strain ATCC BAA-918 / JCM 12380 / KOD1)}; Related PDB entries: 5K2M_F 5K2M_N 5K2M_E 5K2M_L 5K2M_M	82.62	0.95	1.7	47	53

Figure S3E

Chromo-J3C

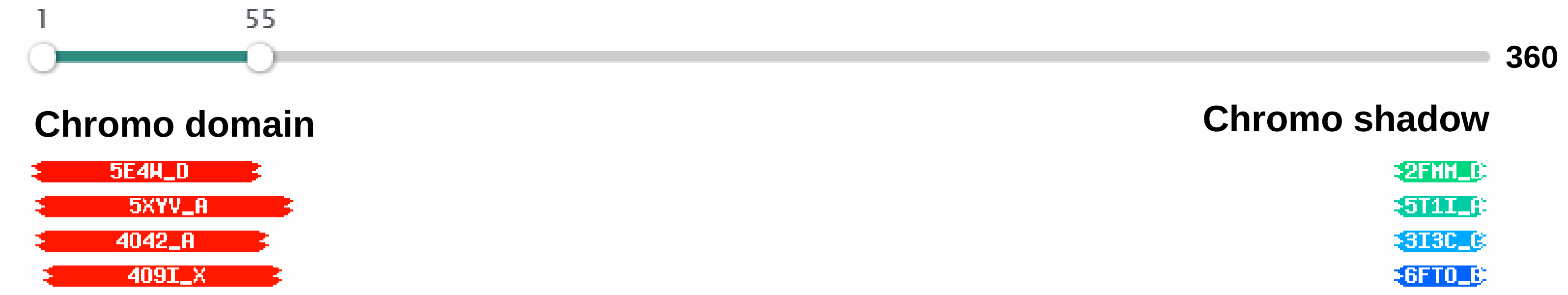

Chromo domain

Hit	Name	Probability	E-value	SS	Cols	Target Length
5E4W_D	Thioredoxin-1, Signal recognition particle 43; Signal recognition particle, chromodomain, membrane; HET: GOL; 2.8A {Escherichia coli O157:H7}; Related PDB entries: 5E4W_C	98.52	1.1e-7	4.2	49	105
5XYV_A	RHINO, Protein deadlock; piRNA pathway, Chromoshadow, Complex, PROTEIN; 2.1A {Drosophila melanogaster}; Related PDB entries: 5XYV_B	98.07	0.0000015	1.4	58	78
4O42_A	Chromodomain-helicase-DNA-binding protein 1 (E.C.3.6.4.12), Nonstructural; viral, chromodomain, Structural Genomics, Structural; HET: MLY, UNX; 1.87A {Homo sapiens}; Related PDB entries: 4NW2_A 4NW2_C 2B2V_B 2B2V_A 2B2W_B 2B2W_A 2B2T_B 2B2T_A 2B2U_B 2B2Y_B 2B2U_A 2B2Y_A	97.99	0.0000054	3.6	54	194
4O9I_X	Chromodomain-helicase-DNA-binding protein 4 (E.C.3.6.4.12); CHD4 double chromodomains, HYDROLASE; HET: MSE; 2.6A {Homo sapiens}	97.84	0.0000097	2.6	57	202

Chromo shadow

2FMM_D	Protein EMSY, Chromobox protein homolog; ENT DOMAIN, CHROMO SHADOW DOMAIN; HET: SO4; 1.8A {Homo sapiens} SCOP: b.34.13.2; Related PDB entries: 1S4Z_B 1S4Z_A 2FMM_B 2FMM_A 2FMM_C 1DZ1_A 1DZ1_B	92.35	0.066	1	52	74
5T1I_A	Chromobox protein homolog 3, histone-H3; Structural Genomics, Structural Genomics Consortium; HET: UNX, N7P, AAR; 1.6A {Homo sapiens}; Related PDB entries: 5T1I_B 6HW2_C 3KUP_B 3KUP_D 3KUP_A 3KUP_C	60.15	7.8	2.1	21	68
3I3C_C	Chromobox protein homolog 5; CBX5, Chromo Shadow Domain, Structural; 2.48A {Homo sapiens} SCOP: b.34.13.2; Related PDB entries: 3I3C_B 3I3C_D 3I3C_A	54.34	12	2.3	21	75
6FTO_B	Chromo domain-containing protein 2, Chromatin; chromoshadow domain, complex, chromatin remodeler; HET: HEZ; 1.6A {Schizosaccharomyces pombe}; Related PDB entries: 6FTO_A	48.38	19	2.5	21	66

Figure S3F

CS-J3C

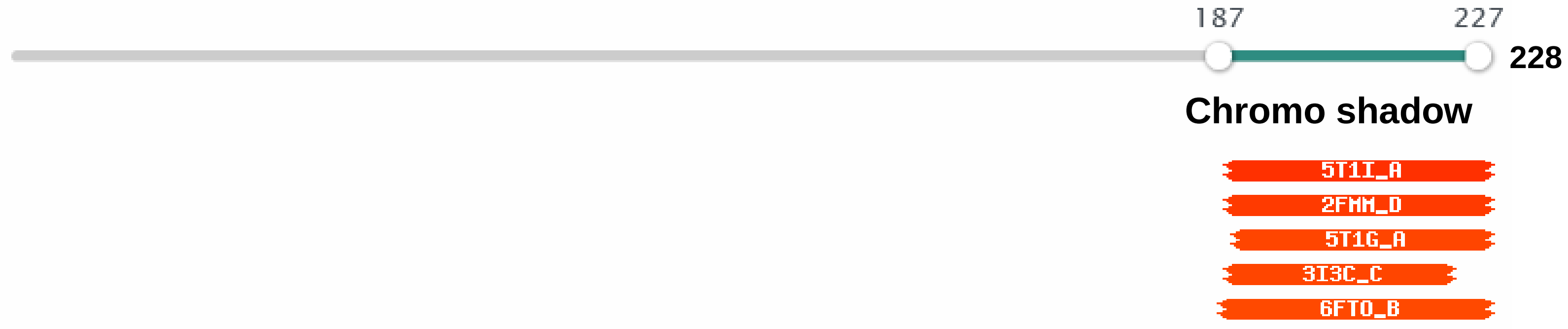

Chromo shadow

Hit	Name	Probability	E-value	SS	Cols	Target Length
5T1I_A	Chromobox protein homolog 3, histone-H3; Structural Genomics, Structural Genomics Consortium; HET: UNX, N7P, AAR; 1.6A {Homo sapiens}; Related PDB entries: 5T1I_B 6HW2_C 3KUP_B 3KUP_D 3KUP_A 3KUP_C	95.88	0.011	3.3	41	68
2FMM_D	Protein EMSY, Chromobox protein homolog; ENT DOMAIN, CHROMO SHADOW DOMAIN; HET: SO4; 1.8A {Homo sapiens} SCOP: b.34.13.2; Related PDB entries: 1S4Z_B 1S4Z_A 2FMM_B 2FMM_A 2FMM_C 1DZ1_A 1DZ1_B	95.12	0.027	3.2	41	74
5T1G_A	Chromobox protein homolog 1, H31; chromo shadow domain, structural genomics; 1.9A {Homo sapiens}; Related PDB entries: 3Q6S_B 3Q6S_A 3Q6S_D 3Q6S_C 6HW2_B	94.18	0.061	3.1	40	79
3I3C_C	Chromobox protein homolog 5; CBX5, Chromo Shadow Domain, Structural; 2.48A {Homo sapiens} SCOP: b.34.13.2; Related PDB entries: 3I3C_B 3I3C_D 3I3C_A	94.16	0.059	3.1	35	75
6FTO_B	Chromo domain-containing protein 2, Chromatin; chromoshadow domain, complex, chromatin remodeler; HET: HEZ; 1.6A {Schizosaccharomyces pombe}; Related PDB entries: 6FTO_A	93.78	0.086	3.2	42	66

Figure S3G

HPC-J3C (Tb927.4250)

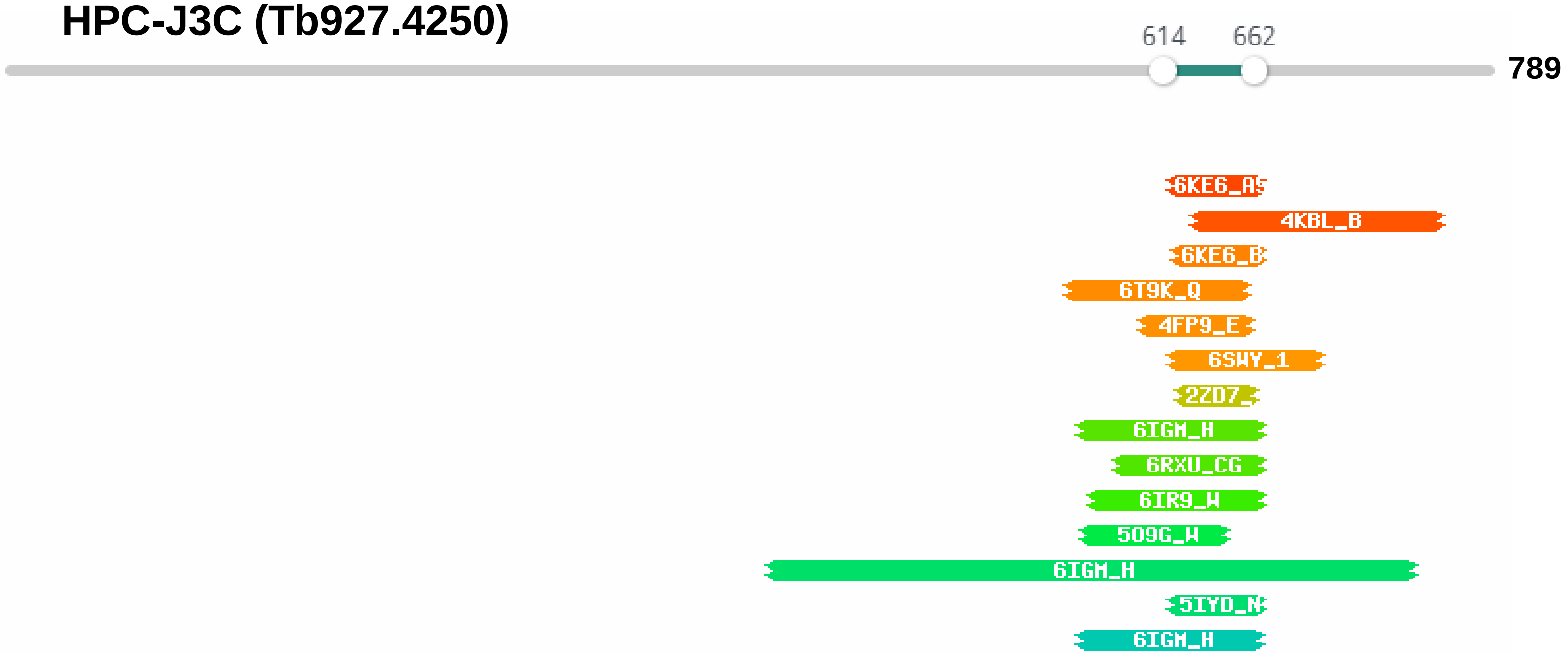

Nr	Hit	Name	Probability	E-value	Score	SS	Aligned cols	Target Length
1	6KE6_A5	40S ribosomal protein S1-A, 40S; ribosome assembly, 90S pre-ribosome, cryo-EM; HET: GTP; 3.4A {Saccharomyces cerevisiae	94.15	0.047	55.57	3.4	49	643
2	4KBL_B	E3 ubiquitin-protein ligase ARIH1 (E.C.6.3.2.-); RING-IBR-RING, E3 ubiquitin ligase, LIGASE; HET: ZN; 3.3A {Homo sapiens	92.76	0.22	50.65	5.6	91	559
3	6KE6_B1	40S ribosomal protein S1-A, 40S; ribosome assembly, 90S pre-ribosome, cryo-EM; HET: GTP; 3.4A {Saccharomyces cerevisiae	88.99	0.36	50.97	3	47	923
4	6T9K_Q	Transcription factor SPT20, Protein SPT3; Coactivator, Transcription, Histone acetyltransferase, Histone; 3.3A {Saccharo	88.17	1.2	50.31	6.5	76	657
5	4FP9_E	Putative methyltransferase NSUN4 (E.C.2.1.1.-), mTERF; Modification enzyme, TRANSFERASE; HET: SAM, SO4; 2.9A {Homo sapie	87.83	0.88	42.65	4.4	59	335
6	6SWY_1	Vacuolar import and degradation protein; Suppressed, Suppreseed, LIGASE; 3.2A { Saccharomyces cerevisiae YJM1133}	87.23	0.92	52.23	5	79	1064
7	2ZD7_A	Vacuolar protein sorting-associated protein 75; Histone chaperone, vps75, NAP1, Nucleus; 1.85A {Saccharomyces cerevisiae	81.27	1.8	40.93	3.4	41	264
8	6IGM_H	RuvB-like 1 (E.C.3.6.4.12), RuvB-like 2; SRCAP complex, TRANSCRIPTION; 4.0A {Homo sapiens}	75.06	2.7	53.85	3.3	97	3230

Figure S3H

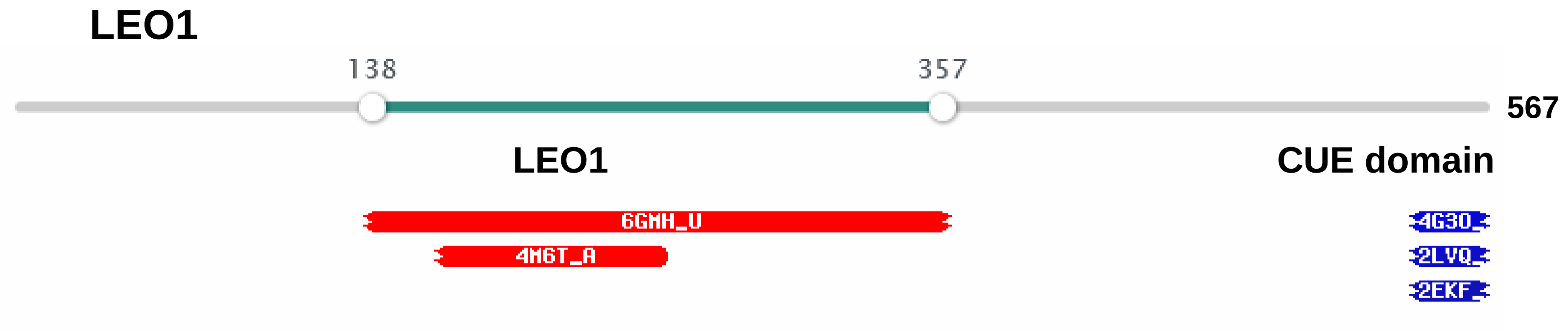

LEO1

Hit	Name	Probability	E-value	SS	Cols	Target Length
6GMH_U	RPB1, DNA-directed RNA polymerase subunit; DNA, RNA Polymerase, DSIF, PAF1c; HET: SEP, TPO; 3.1A {Sus scrofa}	99.97	8.1e-31	13.2	188	776
4M6T_A	RNA polymerase II-associated factor 1; Paf1-Leo1 subcomplex, transcription elongator, Transcription; HET: SAM; 2.498A {Homo sapiens}	99.85	2e-21	8.3	81	183

CUE

4G3O_A	--REMARK 3; all-helical structure, ligase, BAG6; 1.6A {Homo sapiens}; Related PDB entries: 2EJS_A	37.21	68	4.2	28	58
2LVQ_D	Ubiquitin, E3 ubiquitin-protein ligase AMFR; CUE domain, SIGNALING PROTEIN-LIGASE complex; NMR {Homo sapiens}; Related PDB entries: 2LVN_C 2LVO_C 2LVP_C	35.75	77	4.2	28	52
2EKF_A	Ancient ubiquitous protein 1; CUE, Ubiquitin ligase complex, Ubiquitin-conjugating; NMR {Homo sapiens}	34.55	80	4.3	28	61

Figure S3I

CDC73

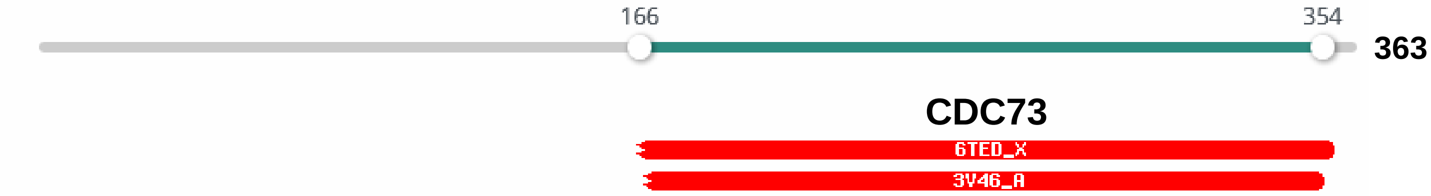

CDC73

Hit	Name	Probability	E-value	SS	Cols	Target Length
6TED_X	DNA-directed RNA polymerase subunit, DNA-directed; Polymerase, elongation complex, RNA, DNA; HET: SEP, TPO;{Sus scrofa}	100	2.2e-43	17.7	175	531
3V46_A	Cell division control protein 73; Ras-like fold, non-GTP binding, Protein; 1.549A {Saccharomyces cerevisiae}; Related PDB entries: 4DM4_A 4DM4_B	100	3.4e-43	16.4	160	170

Figure S3J

CTR9

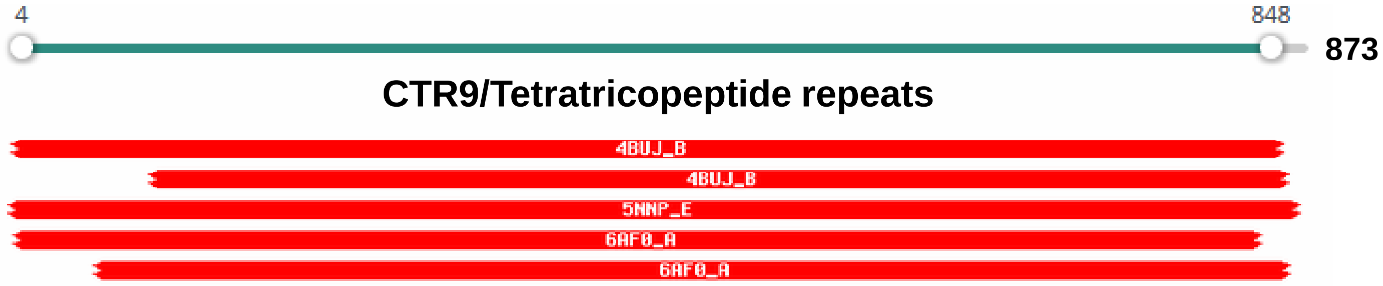

CTR9/Tetratricopeptide repeats

Hit	Name	Probability	E-value	SS	Cols	Target Length
4BUJ_B	ANTIVIRAL HELICASE SKI2 (E.C.3.6.4.13), SUPERKILLER; HYDROLASE, DEXH BOX HELICASE, RNA; HET: SO4; 3.7A {SACCHAROMYCES CEREVISIAE}; Related PDB entries: 4BUJ_F 5MC6_i	100	1.9e-44	89.5	784	1436
4BUJ_B	ANTIVIRAL HELICASE SKI2 (E.C.3.6.4.13), SUPERKILLER; HYDROLASE, DEXH BOX HELICASE, RNA; HET: SO4; 3.7A {SACCHAROMYCES CEREVISIAE}; Related PDB entries: 4BUJ_F 5MC6_i	100	1.7e-41	77.7	695	1436
5NNP_E	N-terminal acetyltransferase-like protein, Naa10, HypK; N-acetylation, NATs, Naa15, Naa10, HypK; HET: CMC, PO4, FME, GOL; 2.602A {Chaetomium thermophilum (strain DSM 1495 / CBS 144.50 / IMI 039719)}; Related PDB entries: 5NNR_A 5NNR_D 5NNP_A	100	3.1e-41	66.3	693	745
6AF0_A	Ctr9 protein, Paf1 protein, Cdc73; Transcription elongation Paf1 ; 2.88A {Myceliophthora thermophila (strain ATCC 42464 / BCRC 31852 / DSM 1799)}	100	8.4e-39	85.2	735	939
6AF0_A	Ctr9 protein, Paf1 protein, Cdc73; Transcription elongation Paf1 ; 2.88A {Myceliophthora thermophila (strain ATCC 42464 / BCRC 31852 / DSM 1799)}	100	9.8e-38	76.1	675	939

Figure S3K

DCNL

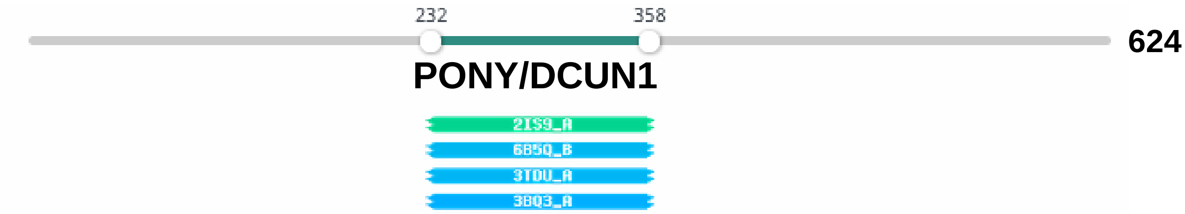

Hit	Name	Probability	E-value	SS	Cols	Target Length
2IS9_A	Defective in cullin neddylation protein; ubiquitin, DCN1, TRANSCRIPTION; HET: MES; 1.92A {Saccharomyces cerevisiae}; Related PDB entries: 3O6B_I 3O6B_A 3O6B_C 3O6B_E 3O6B_G 3O2P_A 3TDI_B 3TDI_A	61.6	64	9.1	102	204
6B5Q_B	DCN1-like protein 1; E3 ligase, complex, Ligase-Inhibitor complex; HET: MLY, PPI, CZS, 2KY, PGE, 1XY; 2.16A {Homo sapiens}; Related PDB entries: 5UFI_B 5UFI_D 6B5Q_A 5UFI_A 5UFI_C	55.91	43	7	105	225
3TDU_A	DCN1-like protein 1, Cullin-1, NEDD8-conjugating; E2:E3, Ligase-protein binding complex; 1.5A {Homo sapiens}; Related PDB entries: 4GAO_B 4GAO_A 4GAO_D 4GAO_G 4P5O_F 3TDZ_B 3TDZ_A 4P5O_E 3TDU_B	55.56	39	6.6	105	200
3BQ3_A	Defective in cullin neddylation protein; ubiquitin, Nedd8, neddylation, ubiquitination, SCF; HET: MSE; 1.9A {Saccharomyces cerevisiae}	54.65	71	8.4	102	270

Figure S3L

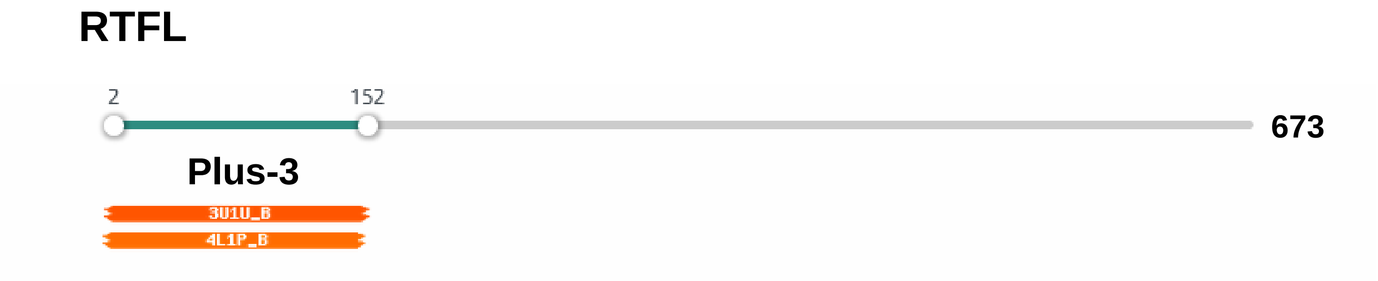

Hit	Name	Probability	E-value	SS	Cols	Target Length
3U1U_B	RNA polymerase-associated protein RTF1 homolog; PLUS-3, transcription elongation, Structural Genomics; HET: SO4, MSE, UNX; 1.8A {Homo sapiens} SCOP: b.34.21.1; Related PDB entries: 3U1U_A	92.84	0.048	1	109	137
4L1P_B	RNA polymerase-associated protein RTF1 homolog; Tutor, Plus3, Peptide binding protein; HET: GOL; 2.12A {Homo sapiens} SCOP: l.1.1.1, b.34.21.1; Related PDB entries: 2BZE_A 2DB9_A 4L1U_B 4L1U_F 4L1U_A 4L1U_D 4L1U_C 4L1U_E 4L1P_A	90.86	0.095	0.6	108	138
