# Supplementary material for: Chromatin-Associated Protein Complexes Link DNA Base J and Transcription Termination in Leishmania
Source: mSphere. 2021 Feb 24;6(1):e01204-20. doi: 10.1128/mSphere.01204-20 (PMC8544896; doi:10.1128/mSphere.01204-20)
Supplement: TABLE S2 [file msphere.01204-20-st002.pdf]

Table S2. Top 50 genes with increased mRNA levels after depletion of JBP3.

Gene ID	Description	Location			Ratio	
		Chr	Start	locus type	log_2 FC	p-value
LtaP35.2610	hypothetical protein	35	949942	cTTS	2.007	0.000
LtaP05.1050	3-mercaptopyruvate sulfurtransferase	05	368927	cTTS-Cen	2.003	0.000
LtaP29.1570	hypothetical protein, conserved	29	628209	cTTS	1.748	0.000
LtaP33.1920	OTT_1508-like deaminase, putative	33	720048	cTTS	1.690	0.000
LtaP14.0450	UDP-glucoronosyl and UDP-glucosyl transferase, putative	14	151694	cTTS-Cen	1.429	0.000
LtaP29.1550	RNA binding protein, putative	29	611570	cTTS	1.232	0.000
LtaP09.1040	hypothetical protein	09	411612	cTTS	1.195	0.000
LtaP36.4670	related to elongation factor-2 kinase efk-1b isoform-like protein	36	1751185	PTU-int	1.191	0.000
LtaP25.1540	hypothetical protein	25	582621	uTTS-Cen	1.185	0.000
LtaP25.1540	hypothetical protein	25	584002	(uTTS-Cen)	1.170	0.000
LtaP32.1470	hypothetical protein, conserved	32	539202	cTTS	1.099	0.000
LtaP36.5010	Sugar efflux transporter for intercellular exchange, putative	36	1876836	cTTS	1.085	0.000
LtaP28.1470	Zinc finger C-x8-C-x5-C-x3-H type (and similar), putative	28	532778	PTU-int	1.085	0.000
LtaP33.1920	hypothetical protein	33	722447	cTTS	1.065	0.000
LtaP28.0390	Sugar efflux transporter for intercellular exchange, putative	28	117321	cTTS	1.059	0.000
LtaP35.2810	hypothetical protein, conserved	35	1022077	PTU-int	1.040	0.000
LtaP36.4990	hypothetical protein, conserved	36	1869851	cTTS	1.034	0.000
LtaP32.1660	PSP1 C-terminal conserved region containing protein, putative	32	608524	PTU-int	0.988	0.000
LtaP25.1540	hypothetical protein	25	583642	(uTTS-Cen)	0.980	0.000
LtaP35.2810	hypothetical protein, conserved	35	1024004	PTU-int	0.971	0.000
LtaP08.0570	hypothetical protein, conserved	08	227029	PTU-int	0.947	0.000
LtaP35.2800	hypothetical protein	35	1016026	PTU-int	0.944	0.000
LtaP20.0810	phosphopantetheinyl transferase-like protein	20	312598	uTSS	0.939	0.000
LtaP35.2580	cytochrome P450 reductase, putative	35	939061	(cTTS)	0.929	0.000
LtaP31.2880	hypothetical protein, conserved	31	1196740	PTU-int	0.919	0.000
LtaP14.0440	cystathionine beta-lyase-like protein	14	148441	PTU-int	0.907	0.000
LtaP08.0680	hypothetical protein, conserved	08	289181	cTTS	0.907	0.000
LtaP28.2750	hypothetical protein, conserved	28	1035293	cTTS	0.895	0.000
LtaP27.2430	hypothetical protein, conserved	27	1067913	rRNA locus	0.875	0.000
LtaP27.0920	Protein of unknown function (DUF778), putative	27	371868	cTTS	0.866	0.000
LtaP31.0590	hypothetical protein	31	199619	cTTS	0.865	0.000
LtaP26.1420	hypothetical protein	26	523787	PTU-int	0.841	0.000
LtaP36.4970	Leucine Rich repeat, putative	36	1863365	((cTTS))	0.839	0.000
LtaP26.2430	nitrilase, putative	26	884318	uTSS	0.829	0.000
LtaP35.2610	DHHC palmitoyltransferase, putative	35	947686	(cTTS)	0.822	0.000
LtaP35.2690	Heat shock factor binding protein 1, putative	35	967052	((uTTS))	0.801	0.000
LtaP33.1440	hypothetical protein, conserved	33	522812	uTTS	0.786	0.000
LtaP30.2110	alcohol dehydrogenase, putative	30	758540	cTTS	0.782	0.000
LtaP18.1620	hypothetical protein, conserved	18	707990	(tTTS)	0.778	0.000
LtaP35.1100	aldose 1-epimerase, putative	35	330066	PTU-int	0.774	0.000
LtaP10.0470	Leishmanolysin, putative	10	496579	(cTTS)	0.767	0.000
LtaP27.0930	Pep3/Vps18/deep orange family/Region in Clathrin and VPS/Vacuolar sorting protein 39 domain 2, putative	27	373503	cTTS	0.764	0.000
LtaP27.1780	Phosphoglycerate kinase, putative	27	716788	(cTTS)	0.758	0.000
LtaP32.1700	AAA domain/PIF1-like helicase, putative	32	621326	PTU-int	0.752	0.000
LtaP23.0680	oxidoreductase-like protein	23	210491	uTSS	0.750	0.000
LtaP32.1720	hypothetical protein, conserved	32	631137	PTU-int	0.748	0.000
LtaP32.1380	Protein tyrosine kinase/Protein kinase domain containing protein, putative	32	502863	PTU-int	0.747	0.000
LtaP31.2410	hypothetical protein	31	984533	(uTTS)	0.746	0.000
LtaP28.0350	hypothetical protein	28	111770	cTTS	0.744	0.000
LtaPcontig283-1	hypothetical protein, conserved	31	263007	PTU-int	0.740	0.000
