# Supplementary material for: Chromatin-Associated Protein Complexes Link DNA Base J and Transcription Termination in Leishmania
Source: mSphere. 2021 Feb 24;6(1):e01204-20. doi: 10.1128/mSphere.01204-20 (PMC8544896; doi:10.1128/mSphere.01204-20)
Supplement: TABLE S3 [file msphere.01204-20-st003.pdf]

Table S3. Oligonucleotide primers used for construct creation.

Primer	Sequence (5'-3')
**Primers used for creation of pLEXY-MHTAP**	
MHTAP-BamHI-S	GGATCCCCTAGGGGTACCCAATTGCTCGAGATGGAACAGAAACTGATCTCTG
MHTAP-NotI-AS	GCGGCCGCATGGGCAGGATCAGGTTGAC
**Primers used to generate TAP-tagged proteins**	
HmdUGT-AvrII-ATG	CGGCCTAGGATGTTCTCCCTCAACATCAAG
HmdUGT-SalI-P2790M	CGCGTCGACTGGACCAGGCTCGCCCTC
WD-GT-AvrII-ATG	CGGCCTAGGATGAACAGCTCCCCCGCCCCAAG
WD-GT-SalI-P1161M	CGCGTCGACTGTCTGTCCCTCCTTCAGTGATG
JBP3-AvrII-M9P	CGGCCTAGGCTTCTCAGCATGTCCTCAAAAC
JBP3-XhoI-P1989M	CGGCTCGAGTGTTTGTGAGGACGCCAGGCTC
PP1Ce-AvrII-ATG	CGGCCTAGGATGGCAAGCACGAAAAGAG
PP1Ce-MunI-P1122M	CGGCAATTGGTCGTGGCTCAAAGGATTTG
PNUTS-AvrII-ATG	GCACCTAGGCCATGAGCGTGGAGGAACTG
PNUTS-XhoI-P796M	GCACTCGAGCATGGGGATGGGGAGGGGCTC
LEO1-AvrII-ATG	GCACCTAGGTGGAATGGAGTGCCAAGCAG
LEO1-SalI-P1701M	GCAGTCGACCAGCTCACCAGGAAACAGTG
Chromo-J3C-AvrII-ATG	GCACCTAGGATGACCTACTACACCGTTGAG
Chromo-J3C-XhoI-P1080M	GCACTCGAGATGCAGAACGACCGAGTTC
CS-J3C-AvrII-ATG	GCACCTAGGATGCACGACGATCAGCTGCGCTTC
CS-J3-SalI-P684M	GCAGTCGACCGCCCAAACAGACATCGAGAG
**Primers used for CRISPR/Cas9 deletions**	
JBP3-up	**TGTGCACTACCCACCGTCTTTAGAACTCGG**GTATAATGCAGACCTGCTGC
JBP3-down	**CTTCCTCCGTGGCCCTCATGTGCGACTCCT**CCAATTTGAGAGACCTGTGC
JBP3-M84P	ACCCACCGTCTTTAGAACTC
JBP3-P2147M	TCTCTGATGGGCGTACATTC
